# Electrophysiological aftereffects of high-frequency transcranial random noise stimulation (hf-tRNS): an EEG investigation

**DOI:** 10.1007/s00221-021-06142-4

**Published:** 2021-06-08

**Authors:** Filippo Ghin, Louise O’Hare, Andrea Pavan

**Affiliations:** 1 School of Psychology, University of Lincoln, Brayford Wharf East, Lincoln, LN5 7AY UK; 2 Cognitive Neurophysiology, Department of Child and Adolescent Psychiatry, Faculty of Medicine of the TU Dresden, Fetscherstraße 74, Schubertstraße 42, 01309 Dresden, Germany; 3 Division of Psychology, Nottingham Trent University, 50 Shakespeare Street, Nottingham, NG1 4FQ UK; 4 Department of Psychology, University of Bologna, Viale Berti Pichat, 5, 40127 Bologna, Italy

**Keywords:** High-frequency transcranial random noise stimulation (hf-tRNS), Transcranial electrical stimulation, Visual evoked potentials, Resting state, Time–frequency analysis, Global motion

## Abstract

There is evidence that high-frequency transcranial random noise stimulation (hf-tRNS) is effective in improving behavioural performance in several visual tasks. However, so far there has been limited research into the spatial and temporal characteristics of hf-tRNS-induced facilitatory effects. In the present study, electroencephalogram (EEG) was used to investigate the spatial and temporal dynamics of cortical activity modulated by *offline* hf-tRNS on performance on a motion direction discrimination task. We used EEG to measure the amplitude of motion-related VEPs over the parieto-occipital cortex, as well as oscillatory power spectral density (PSD) at rest. A time–frequency decomposition analysis was also performed to investigate the shift in event-related spectral perturbation (ERSP) in response to the motion stimuli between the pre- and post-stimulation period. The results showed that the accuracy of the motion direction discrimination task was not modulated by *offline* hf-tRNS. Although the motion task was able to elicit motion-dependent VEP components (P1, N2, and P2), none of them showed any significant change between pre- and post-stimulation. We also found a time-dependent increase of the PSD in alpha and beta bands regardless of the stimulation protocol. Finally, time–frequency analysis showed a modulation of ERSP power in the hf-tRNS condition for gamma activity when compared to pre-stimulation periods and Sham stimulation. Overall, these results show that *offline* hf-tRNS may induce moderate aftereffects in brain oscillatory activity.

**Supplementary Information:**

The online version contains supplementary material available at 10.1007/s00221-021-06142-4.

## Introduction

High-frequency transcranial random noise stimulation (hf-tRNS) is a non-invasive brain stimulation technique which has been shown to improve the performance in a range of visual and cognitive tasks, and is usually delivered online (i.e. during the execution of a specific task) (Campana et al. 2016; Fertonani et al. 2011; Ghin et al. 2018; Pasqualotto 2016; Saiote et al. 2013; Tyler et al. 2018; van der Groen and Wenderoth 2016; van Koningsbruggen et al. 2016). Hf-tRNS delivers alternating current at random intensities and frequencies within specific ranges (e.g. 101–600/640 Hz). On the other hand, *offline* hf-tRNS protocols (i.e. when the stimulation is delivered prior the execution of a task or a measure of interest) have received less attention. For instance, few studies show that *offline* hf-tRNS is able to improve facial processing for expression and emotion (Penton et al. 2017; Romanska et al. 2015; Yang and Banissy 2017) and sustained attention (Harty et al. 2019). However, other studies reported that when delivered *offline*, hf-tRNS failed to induce the same behavioural alterations compared to *online* hf-tRNS (Fertonani et al. 2011; Pirulli et al. 2013). The type of stimulation protocol (i.e. either *online* or *offline*) has been shown to be critical in predicting the modulatory outcomes, which might rely on different neurophysiological mechanisms (Chaieb et al. 2015, 2009; Pirulli et al. 2013). Therefore, it is of fundamental importance to understand the modulatory effects of *offline* hf-tRNS.

Several studies have assessed the effects of hf-tRNS to induce medium- and long-term changes in corticospinal excitability. These studies delivered the stimulation over the motor cortex and measured the modulation of motor-evoked potentials (MEPs) induced by single-pulse transcranial magnetic stimulation (TMS) delivered at different time points after the electric stimulation. The results showed that hf-tRNS modulated the excitability of the motor cortex by enhancing MEPs, but these effects depended on the duration, intensity, and frequency of the electric stimulation, and on the specific electrode montage (Chaieb et al. 2011; Inukai et al. 2016; Moliadze et al. 2014; Moret et al. 2019; Terney et al. 2008). So far, relatively few studies have investigated the physiological effects of hf-tRNS outside the motor system. Recent studies, recording both EEG and TMS-induced phosphene excitability, showed that hf-tRNS can modulate sensory-related cortical activity (Herpich e 2018; Rufener et al. 2017; Van Doren et al. 2014). For example, Van Doren et al. (2014) administered 20 min of *offlin*e hf-tRNS stimulation bilaterally over the temporal cortex; EEG was recorded before and after stimulation. The EEG recording consisted of 5 min of resting state, followed by 7 min of auditory-evoked potentials elicited via an auditory steady-state response (ASSR). The results showed that *offline* hf-tRNS did not modulate resting state activity in all the frequency bands tested (i.e. delta, theta, alpha, beta, low and high gamma). However, *offline* hf-tRNS did modulate ASSR power at around 40 Hz, which is within the low gamma band (33–45 Hz). In addition, Herpich et al. (2018) measured phosphene thresholds using single-pulse TMS after hf-tRNS. Stimulation was delivered bilaterally at 1.0 mA for 20 min over the occipital cortex. The results showed that hf-tRNS was able to increase the excitability of the visual cortex (i.e. lower phosphene thresholds), starting immediately after stimulation and lasting for up to 60 min.

The goal of this study is to extend these preliminary findings and further our knowledge on the underlying cortical mechanisms of *offline* hf-tRNS, and how it modulates the activity of the visual cortex. To this purpose, we investigated hf-tRNS aftereffects by measuring changes on resting state brain oscillations, modulation of the amplitude of visual evoked potentials (VEPs), and modulation of stimulus-locked EEG spectral activity. First, we assessed hf-tRNS aftereffects on resting state brain oscillations. Brain oscillations at rest reflect the general cortical activation state of distinct brain networks and have been linked to specific functions (Groppe et al. 2013; Mantini et al. 2007). The rationale is that if hf-tRNS-induced excitatory effects can outlast the stimulation period this may suggest a temporary modification of the ongoing neural activity at rest. Furthermore, if *offline* hf-tRNS produces long-lasting modulations of visual cortex activity this might be reflected in the neural mechanisms associated with visual processing such as visual evoked potentials (VEPs). Therefore, in this study, we also assessed if *offline* hf-tRNS could modulate VEP amplitude during a motion direction discrimination task. VEPs have been examined to investigate the activation of visual areas associated with motion processing, and distinct VEP components have been often detected in response to moving stimuli (Breveglieri et al. 2013; Miroslav Kuba and Kubová 1992; Kubová et al. 1990; Kubová et al. 1995; Martin et al. 2010; Niedeggen and Wist 1998, 1999). For example, an early positive component identified as P1 has been detected in response to motion-onset stimuli in several studies, although this is also associated with the contrast level of the stimulus (Kubová et al. 1995). A negative component named N2 has been specifically linked to motion processing, and it seems to be generated in extra-striate temporal-occipital and parietal cortical areas (Kuba et al. 2007). A second positive peak named P2 is also found in motion-related VEPs and it seems to be influenced by the complexity of the motion stimulus (Kuba et al. 2007). Finally, we also aim to investigate hf-tRNS-induced aftereffects on the EEG spectral activity during a visual motion task. Specifically, by applying a time–frequency decomposition analysis, we investigated whether *offline* hf-tRNS stimulation could modulate brain activity in the time–frequency domain. Assessing if this stimulation protocol can modulate the brain oscillatory activity is of particular interest, as the effects of hf-tRNS on event-related spectral activity have not been previously investigated. In particular, we will estimate event-related spectral perturbations (ERSP; Makeig 1993; Makeig et al. 2004) by estimating the change in oscillatory power across a range of frequencies using a sliding time window, relative to a pre-stimulus baseline.

The physiological effects of *offline* hf-tRNS over the visual cortex and the extent of its aftereffects on cortical activity are still unclear. Therefore, is important to further investigate whether *offline* hf-tRNS could modulate the neural activity of the visual cortex; while eventual modifications in resting state oscillations might give an indication of the effects of the neurostimulation on the brain at rest, the complementary measures of VEP amplitude and ERSP could give an indication of changes linked to visual stimuli processing and task performance.

## Materials and methods

### Participants

Two of the authors (FG and LOH) and 14 naïve participants took part in the study (7 males, age range 19–33 years). Participants were all right-handed and with normal or corrected to normal visual acuity. Each participant completed a questionnaire to exclude presence of metal objects, heart problems, history of seizures or any neurological disease. Methods were implemented following the World Medical Association Declaration of Helsinki (2013). The present study was approved by the Ethics committee of the University of Lincoln (Project ID: PSY1718268). Written informed consent was obtained from each participant prior the enrolment in the study and they were paid for their time.

### Apparatus

Stimuli were displayed on a 20-inch Iiyama HM204DTA Vision Master Pro Diamontrum U3-CRT monitor with a refresh rate of 85 Hz. Stimuli were generated with Matlab PsychToolbox (Brainard 1997; Kleiner et al. 2007; Pelli 1997). The screen resolution was 1280 × 1024 pixels. Each pixel subtended 1.9 arcmin. A gamma-corrected lookup table (LUT) was used so that luminance was a linear function of the digital representation of the image*.*

### Stimuli

Stimuli were global motion random dot kinematograms (RDKs) made up by 400 white dots (diameter 0.12 deg) presented at the centre of the screen within a circular aperture with a diameter of 12 deg. The Weber contrast of the dots was 0.99. The dots’ density was 3.54 dots/deg^2^. The duration of the RDK was 0.130 s. Dots drifted at a speed of 5.04 deg/s and had a limited lifetime of 47 ms. Dots appeared asynchronously on the display and had an equal probability of being selected as either signal or noise dots (Morgan and Ward 1980; Newsome and Paré 1988). Any dot exceeding the limited lifetime was replaced by a new dot at a different randomly selected position within the circular window. In addition, dots that moved outside the circular window were replaced by a new dot at a different randomly location within the circular window, thus maintaining the same density. Signal dots were constrained to move globally either leftward or rightward, whereas noise dots moved in random directions.

### Transcranial random noise stimulation

**Fig. 1:**
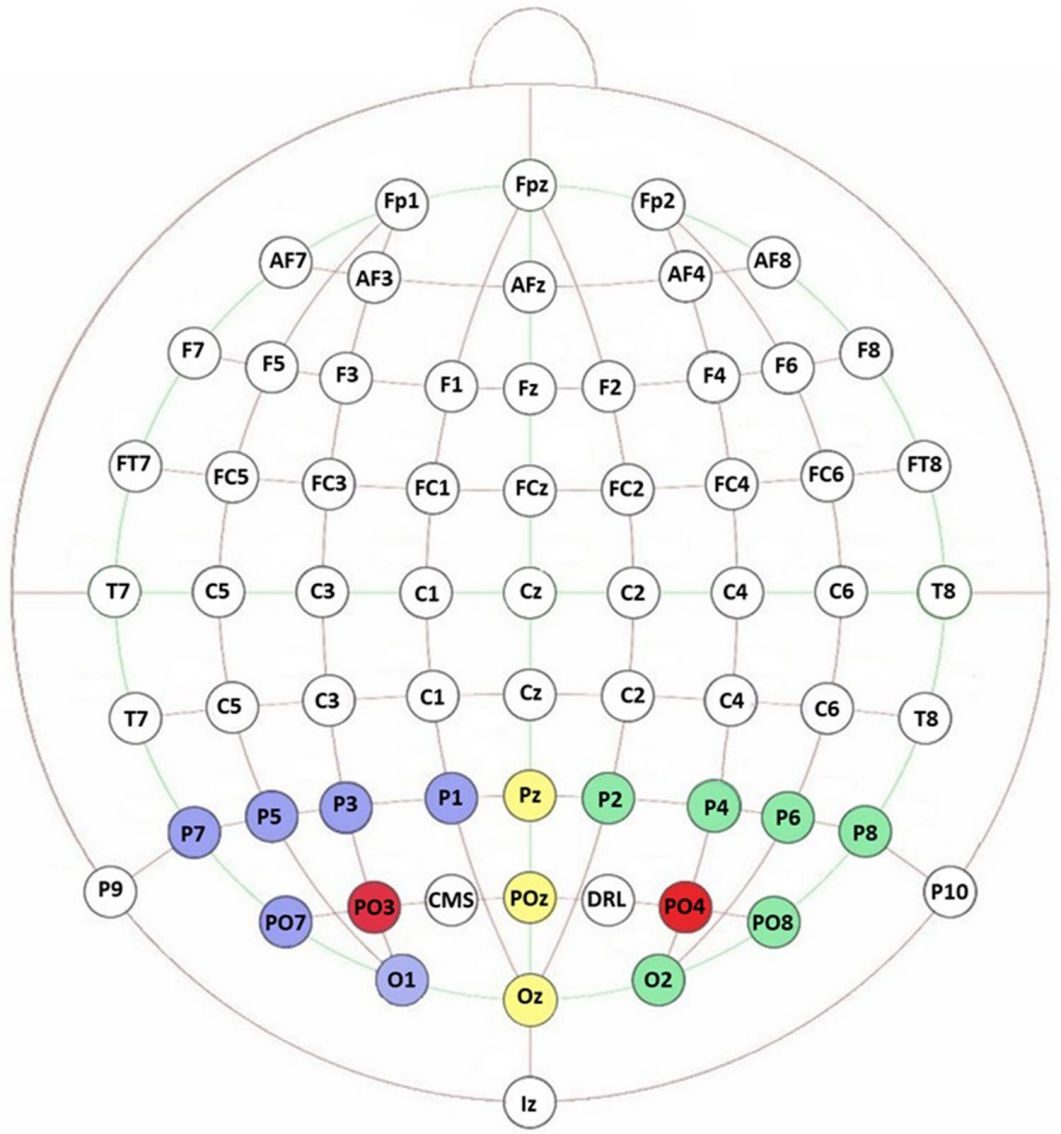
Representation and localization of the electrodes of interest. Electrode selected were Pz, POz, Oz, O1, P7, P5, P3, P1, PO7, P2, P4, P6, P8, PO8, and O2 following the 10–20 system. Electrodes were divided in three main Regions: Left (blue), Central (yellow) and Right (green). The red circles illustrate the location of the hf-tRNS and Sham electrodes that occupied the PO3 and PO4 positions

Stimulation was delivered by a battery driven stimulator (BrainSTIM, EMS; http://www.brainstim.it/index.php?lang=en) through a pair of saline–soaked sponge electrodes. The hf-tRNS consisted of alternating current delivered at 1.5 mA with zero offset and applied with random frequencies ranging between 100 and 600 Hz. The intensity of the electrical stimulation was chosen based on previous findings which showed that *online* hf-tRNS delivered at 1.5 mA was able to improve visual motion processing (Ghin et al. 2018), especially when compared with other stimaultion intesites (e.g. 0.5, 0.75, 1.0, and 2.25 mA) (Pavan et al. 2019). There is also evidence that *online* hf-tRNS delivered at 1.5 mA can modulate the cortical activity by reducing the latencies of auditory event-related potentials (Rufener et al. 2017). The total duration of the stimulation was 20 min. Sham stimulation (i.e. control stimulation) was delivered for 30 s at 1.5 mA (Gandiga et al. 2006). The hf-tRNS and Sham stimulation were delivered bilaterally. Electrode position was determined with the 10–20 system, specifically one electrode was placed at the PO3 position while the second electrode was placed at the PO4 (Fig. 1) position, therefore close to the lower portion of the Brodmann’s area 19 which includes the visual area V5 (Siegel and Sapru 2011). This area has been demonstrated to be important for the processing of global motion (Thompson et al. 2009). The two electrodes had an area of 16 cm^2^ and the current density (0.09 mA/cm^2^) was maintained below the safety limits (Bikson et al. 2016; A. Fertonani et al. 2015).

### EEG recording

Recordings were made using a 64-channel Biosemi Active-Two system (BIOSEMI, https://www.biosemi.com/), using Ag–AgCl electrodes. 62 electrodes were positioned using the 10–20 system, with 8 additional electrodes: 2 on the left and right mastoid, 2 infraorbital, 2 suborbital and 2 on the outer canthi of the eye. PO3 and PO4 electrodes were not used as they were replaced by the tES electrodes. EEG signals were first referenced to a common-mode-sense electrode (Metting van Rijn et al. 1991; Metting van Rijn et al. 1990; https://www.biosemi.com/faq/cms&drl.htm) and re-referenced to the linked mastoids offline. Recordings were sampled at 2048 Hz and down sampled to 265 Hz offline.

### Procedure

**Fig. 2:**
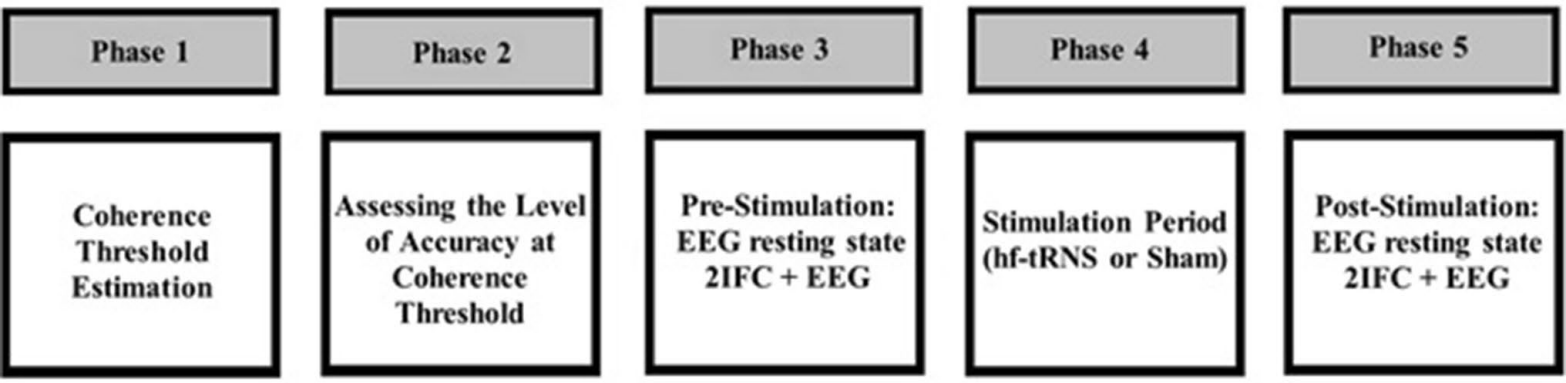
Schematic representation of the five phases of each experimental session

Participants took part in two experimental sessions carried out on two different days. Both sessions had the same procedure. In one session, hf-tRNS was delivered, whereas in the other session, Sham stimulation was delivered. The order of the sessions was randomised across participants. Figure 2 shows the experimental procedure used in the study. Each session consisted of five phases:

### Phase 1: coherence threshold estimation

**Fig. 3:**
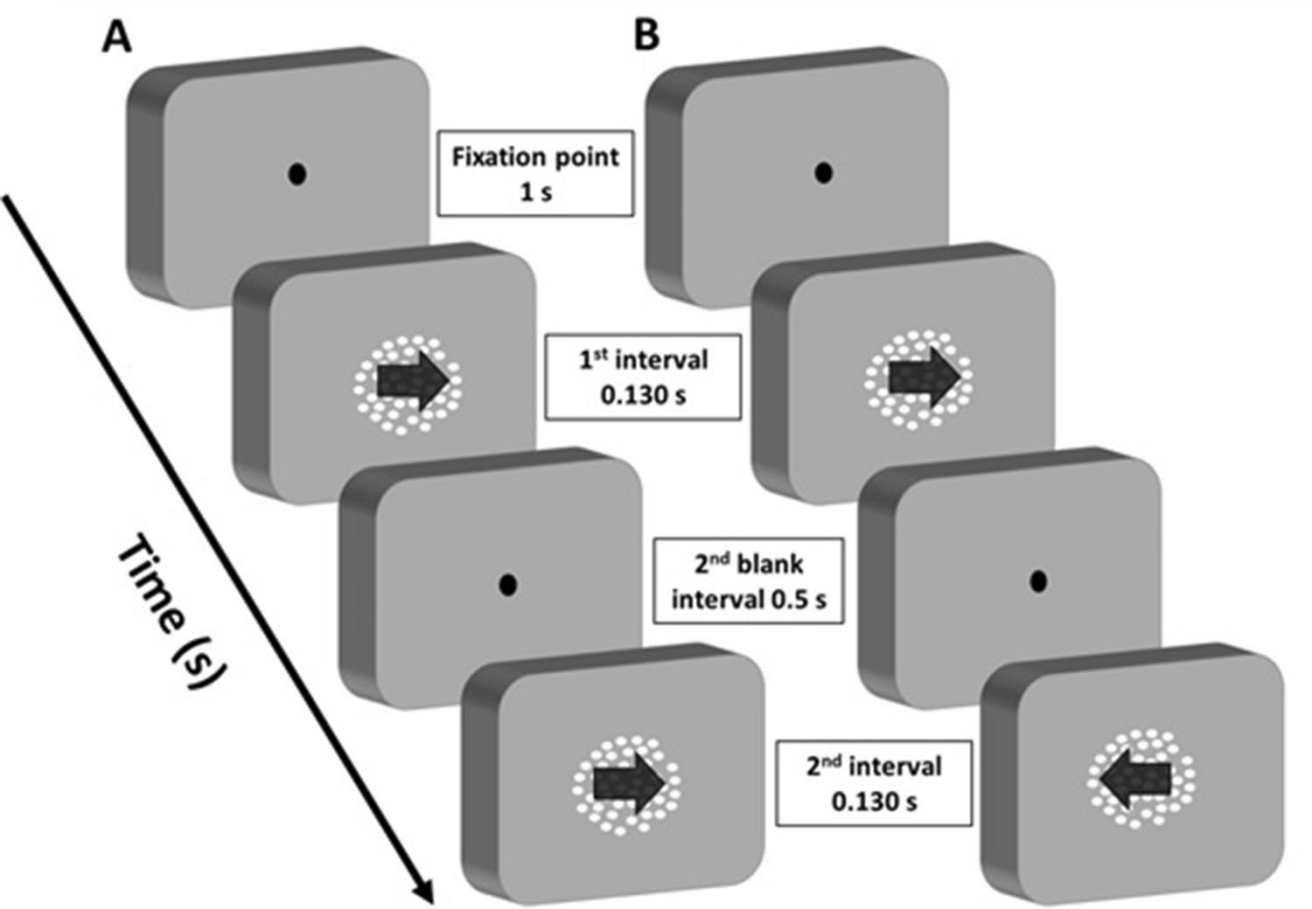
Schematic representation of the motion direction discrimination task. **A** Example of a ‘*same*’ trial, where the RDKs in the two temporal intervals have the same motion direction. **B** Example of a ‘*different*’ trial, where the RDKs have opposite motion directions. The black arrows within the RDKs indicate given stimulus directions in a typical trial. For sake of illustration, they depict motion direction in the figure, but were never presented during the actual experiment

At the beginning of each session, observers performed a two-interval forced choice (2IFC) motion direction discrimination task (Fig. 3) to estimate the individual coherence threshold. The RDKs were presented at the centre of the screen. Participants had to report whether the RDKs presented in the two temporal intervals had the same or different motion direction. Each trial consisted of a fixation point presented for 1 s, followed by two 0.130 s RDKs, with a blank interval of 0.5 s between the two temporal presentations. The inter-trial interval was 1 s. An adaptive MLP staircase (Grassi and Soranzo 2009; Green 1993) was used to track the coherence level producing an accuracy of 80% in motion direction discrimination. This was to generate a strong global motion percept and increase the VEP components normally elicited by a moving stimulus (Kuba et al. 2007; Patzwahl and Zanker 2000). The staircase consisted of 32 trials and participants performed one staircase.

### Phase 2: assessing the level of accuracy at coherence threshold

To accurately estimate the individual coherence threshold producing an accuracy level of 80% in direction discrimination, observers performed the same direction discrimination task at the coherence level estimated in *Phase 1*. The coherence was kept constant across a block of 40 trials. If the resulting accuracy was higher or lower than 80% ± 5%, the observer was asked to perform additional blocks. In these additional blocks, coherence level of the RDKs was manually adjusted by increasing or decreasing the coherence level, on average, in steps of 10 dots (SD = 5 dots). This was repeated until participants reached the desired level of accuracy (80% ± 5%). The coherence level resulting in a performance of 80% ± 5% correct discrimination was used as coherence level for the pre- and post-stimulation conditions.

### Phase 3: pre-stimulation EEG

After EEG and tES setup was completed, EEG was recorded during the resting state (i.e. pre-stimulation EEG). Participants were asked to close their eyes and maintain resting wakefulness while EEG was recorded for 5 min. Immediately after recording the resting state activity, participants were asked to perform five blocks of the 2IFC direction discrimination task while the EEG activity was recorded. The 2IFC task was divided into five blocks to limit fatigue and give the participants the opportunity to rest between blocks (~ 2 min break). The individual coherence level of the RDKs was the one estimated in *Phase 2* and was kept constant across the five experimental blocks. Each block consisted of 40 trials for a total of 200 trials. The 2IFC task lasted approximately 15–20 min. The final accuracy was calculated by averaging the performance values over the five blocks. This phase of the study lasted approximately 20–25 min.

### Phase 4: stimulation period

At the end of the fifth block of the 2IFC task, EEG recording was paused and either hf-tRNS or Sham stimulation was delivered. Observers were unaware of the type of stimulation applied in each session. hf-tRNS was applied for 20 min, whereas the Sham stimulation was applied only for the initial 30 s over a period of 20 min. During the stimulation period, participants remained in the same position, but the light in the room was turned on and participants were asked to relax.

### Phase 5: post-stimulation EEG

In *Phase 5*, the same procedure as the *Phase 3* was implemented. EEG was recorded during 5 min of resting state, followed by other five blocks of the 2IFC motion direction discrimination task. This phase of the study also lasted approximately 20–25 min.

### EEG analysis

Data were analysed using Matlab R2018b (The Mathworks, Natick) and the EEGLAB toolbox (Delorme and Makeig 2004). Data from 62 electrodes were analysed, as PO3 and PO4 were not used since they were replaced by the tES electrodes. Data were bandpass filtered between 0.1 and 40 Hz, after re-referencing to the linked mastoids.

For the analysis of the resting state activity, the 5-min-long recordings were first divided into 2 s epochs. As there was no meaningful pre-stimulus period for these epochs, the whole epoch was used for baseline correction. Power Spectral Density (*PSD*) defined as mean absolute power:

1 $${\text{PSD}}\; = 10{\kern 1pt} \;\log_{10} \left( {\frac{{\mu V^{2} }}{{{\text{Hz}}}}} \right)$$

was estimated for each epoch. This was achieved using the EEGLAB function “Spectopo” with a 1 s Hamming window length and 50% overlap, and then averaged over epochs for each channel. We measured PSD for delta (2–4 Hz), theta (4–8 Hz), alpha (8–14 Hz), and beta (15–30 Hz) bands (Baumgarten et al. 2016; Romei et al. 2008; Spitoni et al. 2013).

Data for the VEPs were collected during the execution of the motion direction discrimination task (i.e. phase 3 and 5 of each experimental session). Data were divided into epochs of 700 ms (− 200 to 500 ms from the each RDK onset). Specifically, each trial of the motion discrimination task was composed of two temporal intervals in which the RDKs were presented (Fig. 3). Therefore, for each trial, we extracted two epochs of 700 ms; one for each RDK presentation. In total we extracted 400 epochs out of the 200 trials in the pre-stimulation EEG phase, and 400 epochs for the post-stimulation EEG phase.

For both resting state period and VEPs, artefacts were removed using the EEGLAB automatic rejection procedure, thus excluding those epochs with fluctuation over ± 100 µV. The Gratton and Coles correction (Gratton et al. 1983) was used to correct for eye movement artefacts (i.e. blinks and saccades). Amplitude criterion for blink detection was set at ± 200 µV over a 20 ms time interval.

Data for both resting state and VEP analysis were averaged across all participants for each Stimulation Type and Recording Time (i.e. pre**-** and post**-**stimulation EEG). For clarity, factors in the analysis are capitalised. Time–frequency analysis was conducted on epochs ranging from − 300 ms before stimulus onset to 500 ms post-stimulus onset. We applied the EEGLAB function *std_precomp()* to remove any ICA component clusters containing artefacts, and used interpolation to estimate the activity for the selected channels (Delorme and Makeig 2004). For each trial two epochs were extracted, one for each RDK temporal interval. Event-related spectral perturbation (ERSP) was estimated using Morlet wavelet filters of 3 cycles for the mother wavelet, for 100 central frequencies evenly spaced between 8 and 75 Hz (Bruns 2004; Smith 2011; Tallon-Baudry et al. 1997). ERSP was estimated for 98 time points with a window size of 417.97 ms. The number of cycles in each wavelet increases linearly with frequency, with 3 cycles at the lowest frequency and 5.63 cycles at the highest frequency.

We restricted the analyses to the electrodes over the sites most relevant to our a-priori hypotheses. In particular, the reason to focus on parieto-occipital areas was based on brain stimulation and neuroimaging studies investigating the neural mechanisms of visual motion processing (Aaen-Stockdale and Thompson 2012; Braddick et al. 2001; Ghin et al. 2018; Herpich et al. 2018; Kuba et al. 2007; Pavan et al. 2019, 2017). In addition, to investigate the effects of *offline* hf-tRNS over the parieto-occipital areas on a motion direction discrimination task, we also selected specific time windows based on previous findings on motion-related VEPs. Specifically, VEPs were defined as the mean response for P1 (70–120 ms; Zalar et al. 2015), N2 (135–180 ms; Kuba and Kubová 1992), and P2 (200–300 ms; Martin et al. 2010) components.

Fifteen electrodes of interest were selected; those surrounding the bilateral electrical stimulation sites: Pz, POz, Oz, O1, P7, P5, P3, P1, PO7, P2, P4, P6, P8, PO8, and O2. Electrodes of interest were grouped in three main regions of interests (ROIs): Left (O1, P1, P3, P5, P7, PO7), Central (OZ, POz, Pz) and Right (O2, P2, P4, P6, P8, PO8) (Fig. 1). This selection was based on two considerations: (i) electrodes of interest were divided according to their position on each hemisphere and on the longitudinal fissure on the parieto-occipital cortex. This is because electrophysiological studies showed that cortical activation from visual motion perception could be asymmetric across the scalp. In fact, left and right hemispheres can show unequal contribution in generating motion-related VEPs (Hollants-Gilhuijs et al. 2000; Niedeggen and Wist 1998; Patzwahl et al. 1994). For example, Kubová et al. (1990) found that presenting horizontally drifting gratings, the 60% of 80 observers tested showed higher N2 amplitude in the right hemisphere with respect to the left one. In addition, Akyuz et al. (2020) using EEG and source localization showed that adaptation to directional motion was dominant in the left hemisphere; (ii) instead of analysing data from single electrodes, we pooled data across groups of electrodes (i.e. for Left, Central and Right regions) to measure the average modulation of cortical activity around the stimulation electrode. This is because the electric field generated by transcranial electrical stimulation can spread beyond the borders of the electrode patch (Datta et al. 2009). Electrodes that did not record any activity were coded as missing electrodes, based on visual inspection of the raw data (i.e. before referencing). For the Central Region, there were two electrodes coded as missing, one in the Sham condition for one participant and one in the hf-tRNS condition for a different participant. More electrodes were missing from the Left Region across the sample, with a mean of 1.8 missing electrodes (SD = 1.03) in the Sham condition, and a mean of 2.9 missing electrodes (SD = 1.22) in the hf-tRNS condition. No electrodes were lost from the Right Region.

## Results

### Behavioural results

**Fig. 4:**
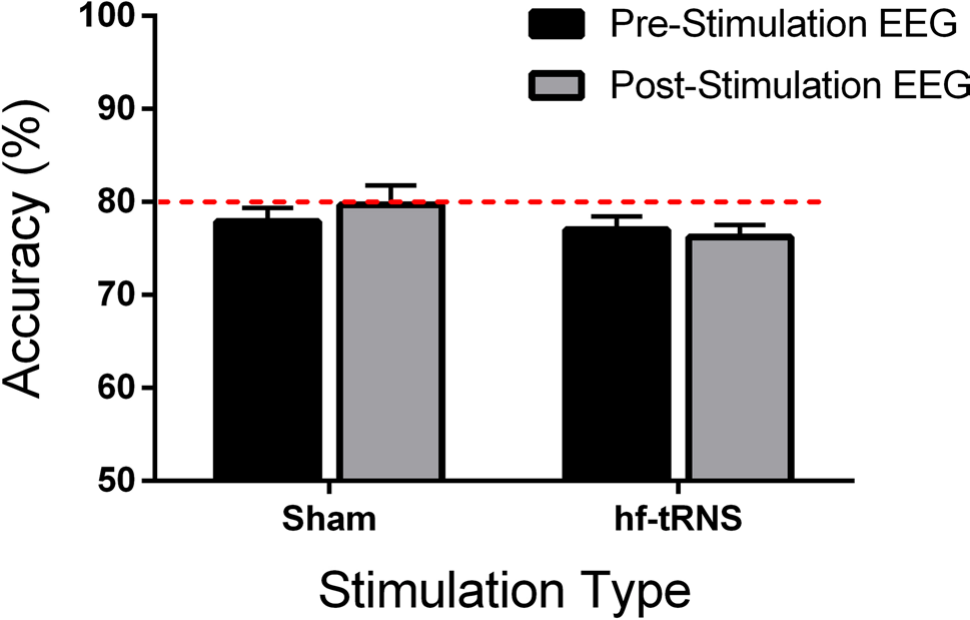
Mean performance values of the motion direction discrimination task measured before (pre-stimulation) and after (post-stimulation) Sham and hf-tRNS. Error bars ± SEM

Figure 4 shows the behavioural results for the motion direction discrimination task between pre- and post-stimulation for Sham and hf-tRNS conditions. A Shapiro–Wilk test reported that residuals were normally distributed in all conditions (*p* > 0.05). A repeated-measures analysis of variance (ANOVA) with Stimulation Type (hf-tRNS and Sham) and Recording Time (pre- and post-stimulation) as within-subject factors was performed. The ANOVA did not reveal any significant effect of Stimulation Type [*F*(1, 15) = 1.564, *p* = 0.230, *Ƞ*^*2*^_*p*_ = 0.094], Recording Time [*F*(1, 15) = 0.698, *p* = 0.416, *Ƞ*^*2*^_*p*_ = 0.044] or interaction between Stimulation Type and Recording Time [*F*(1, 15) = 1.838, *p* = 0.195, *Ƞ*^*2*^_*p*_ = 0.109]. Overall, these results showed that behavioural performance was not influenced by the *offline* hf-tRNS stimulation.

### Electrophysiological results

#### Power spectral density (PSD)

For the PSD estimation, the mean absolute power of the selected electrodes for each observer was extracted. A Shapiro–Wilk test of normality showed that residuals in every condition and Region were normally distributed (*p* > 0.05), except for the Right Region in the post-stimulation EEG in the beta frequency interval (*p* = 0.04). We performed an omnibus repeated-measures ANOVA including Stimulation Type (Sham and hf-tRNS), Recording Time (pre–post-stimulation EEG), Region (Left, Central and Right) and Frequency Band (delta, theta, alpha and beta) as within-subjects factors. The ANOVA reported a significant effect of Recording Time [*F*(1, 15) = 14.767, *p* = 0.002, *Ƞ*^*2*^_*p*_ = 0.496], Region [*F*(2, 30) = 9.593, *p* = 0.001, *Ƞ*^*2*^_*p*_ = 0.390] and Frequency Band [*F*(3, 45) = 325.5, *p* < 0.001, *Ƞ*^*2*^_*p*_ = 0.956], but no significant effect of Stimulation Type [*F*(1, 15) = 0.013, *p* = 0.9, *Ƞ*^*2*^_*p*_ = 0.001]. The four-way interaction Recording Time x Region x Stimulation Type x Frequency Band was not significant [*F*(6,90) = 0.362, *p* = 0.9, *Ƞ*^*2*^_*p*_ =0.024]. The three-way interaction between Recording Time × Region × Stimulation Type was also not significant [*F*(2, 30) = 1.013, *p* = 0.38, *Ƞ*^*2*^_*p*_ = 0.063]. However, we found a significant interaction between Recording Time and Frequency Band [*F*(3, 45) = 7.798, *p* < 0.001, *Ƞ*^*2*^_*p*_ = 0.342], between Region and Frequency Band [*F*(6, 90) = 7.303, *p* < 0.001, *Ƞ*^*2*^_*p*_ = 0.327], and a significant three-way interaction between, Stimulation Type, Region and Frequency Band [*F*(6, 90) = 2.335, *p* = 0.038, *Ƞ*^*2*^_*p*_ = 0.135]. We also conducted separate ANOVAs including as within-subjects factors the Recording Time, ROIs and Stimulation Type separately for each frequency band. We found significant main effects of Recording Time for alpha [*F*(1,16) = 19.722, *p* < 0.001, *Ƞ*^*2*^_*p*_ =0.568] and beta bands [*F*(1,16) = 22.856, *p* < 0.001, *Ƞ*^*2*^_*p*_ =0.604], and main effects of Region for delta [*F*(1,16) = 8.508, *p* = 0.001, *Ƞ*^*2*^_*p*_ =0.362], theta [*F*(1,16) = 17.738, *p* < 0.001, *Ƞ*^*2*^_*p*_ =0.542] and alpha bands [*F*(1,16) = 8.921, *p* = 0.001, *Ƞ*^*2*^_*p*_ =0.373]. Full report of the results is reported in Table S1 of the S*upplementary Material.* The ANOVAs did not report any significant interaction effects (all *p* > 0.05).

To follow-up the results from the omnibus ANOVA, we carried out separate repeated-measures ANOVAs for Frequency Band and Region. In addition, we decided to perform separate ANOVAs for each frequency band because of the power scaling that characterises EEG power data. EEG spectral power falls with increasing frequency, approximately decreasing 1/*f* (where *f* is the frequency). As higher oscillation frequencies are characterised by lower power (i.e. lower amplitude) and vice versa (Cohen 2014), significant differences in PSD between Frequency Bands are expected to occur and a significant difference in PSD between frequency bands would not be informative.

**Fig. 5:**
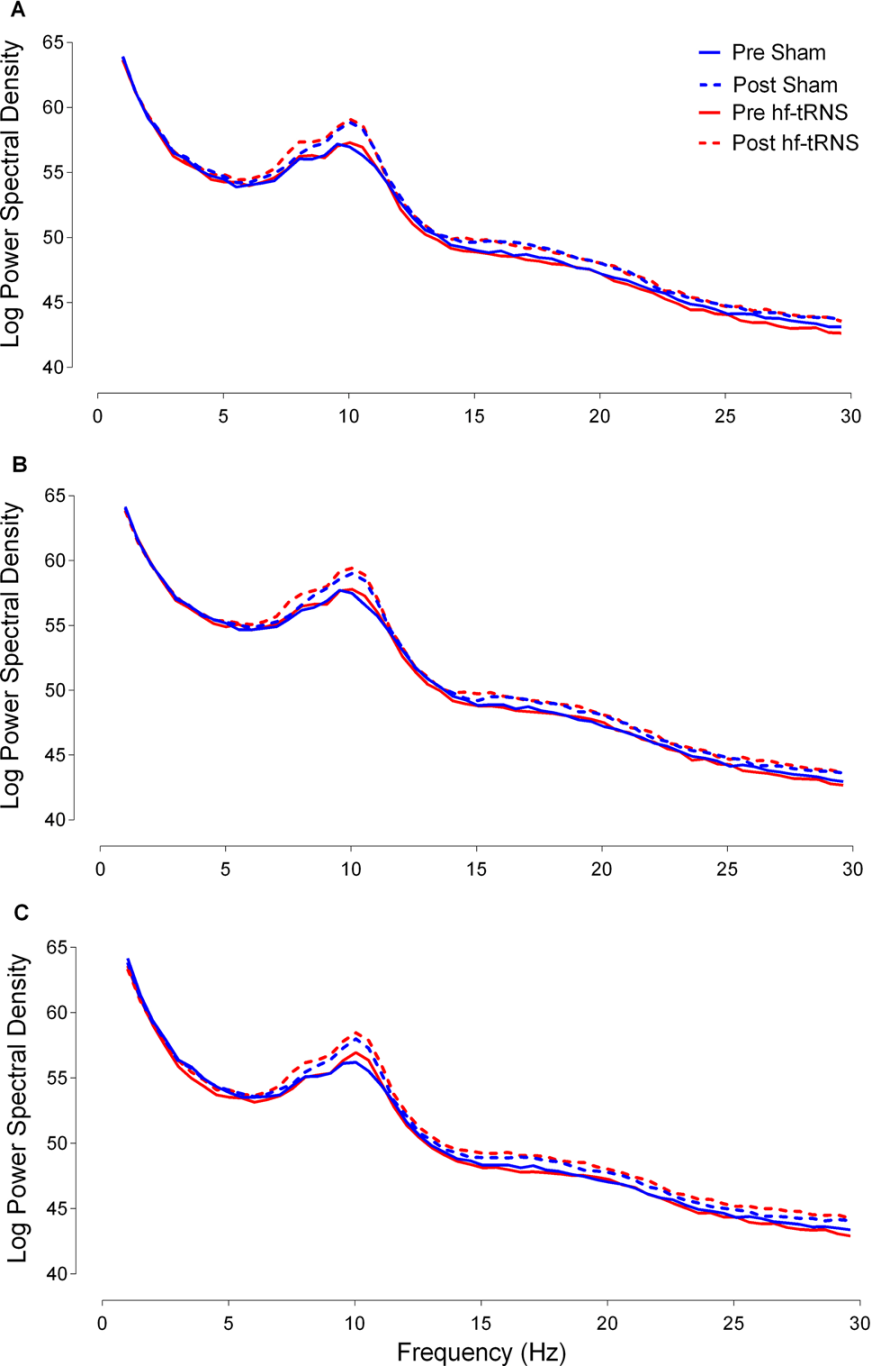
Average power spectra density (PSD) during resting state as a function of frequency (in Hz). Average PSD for hf-tRNS and Sham in the pre-stimulation condition (red and blue continuous lines for hf-tRNS and Sham, respectively) and in the post-stimulation EEG (red and blue dashed lines for hf-tRNS and Sham, respectively). Panel **A**, **B** and C show Left, Central and Right Region, respectively

We performed a repeated-measures ANOVA with Stimulation Type (hf-tRNS and Sham) and Recording Time (pre-stimulation EEG and post-stimulation EEG) as within-subject factors. Separate ANOVAs were conducted for the Left, Central and Right Region and for each Frequency Band: delta (2–4 Hz), theta (4–8 Hz), alpha (8–14 Hz), and beta (15–30 Hz) bands. Figure 5 shows the average PSD for each stimulation condition (hf-tRNS and Sham) and for each Recording time (pre-stimulation EEG and post-stimulation EEG) for the three Regions: Left, Central and Right. The repeated-measures ANOVA for delta and theta bands did not reveal any significant effect of Stimulation Type, Recording Time or interaction between Stimulation Type and Recording Time in any of the three Regions (all *p* > 0.05, see Table 2 *Supplementary Material*).

**Fig. 6:**
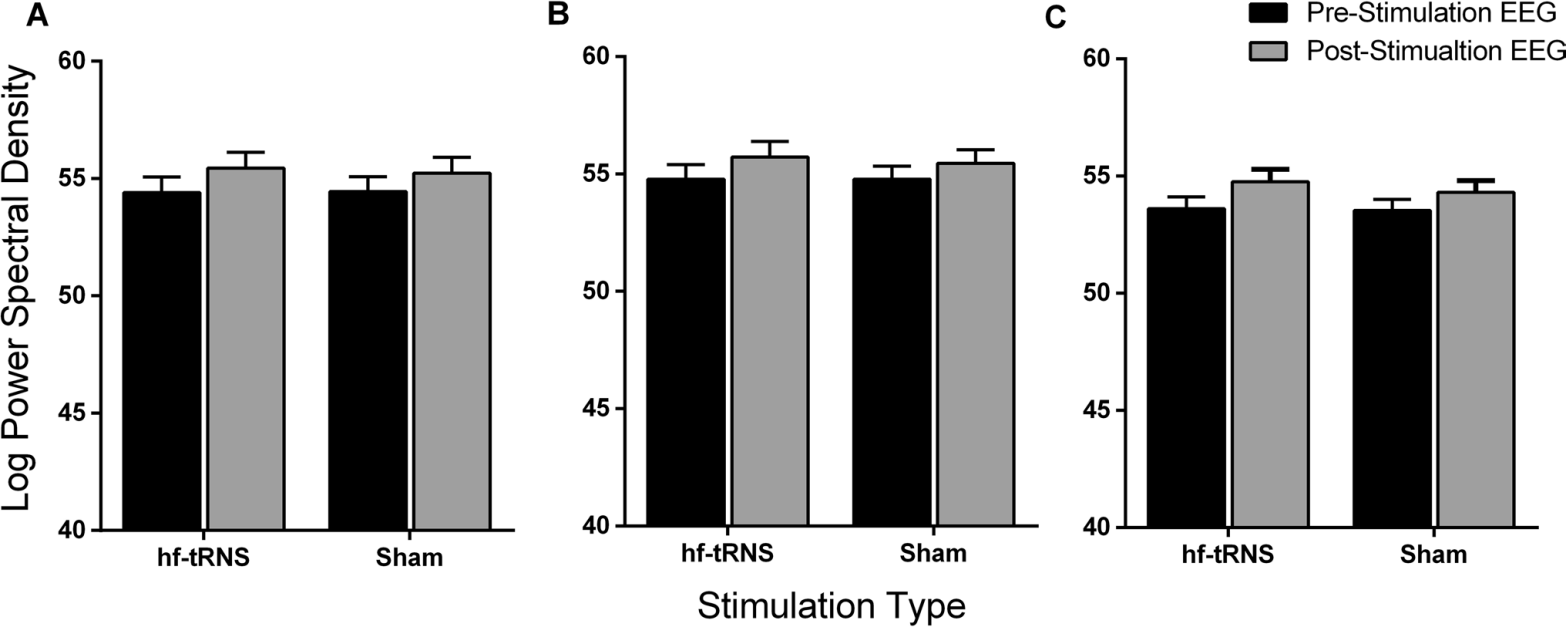
Mean alpha power spectral density (PSD) for Left (**A**), Central (**B**), and Right (**C**) Regions. Each Region includes mean PSD values for hf-tRNS and Sham stimulation for pre-stimulation EEG (black bars) and post-stimulation EEG (grey bars). Error bars ± SEM

However, for alpha and beta bands, the ANOVA showed a different pattern of results. For the alpha oscillations, the ANOVA did not reveal any significant effect of Stimulation Type for the Left [*F*(1, 15) = 0.046, *p* = 0.83, *Ƞ*^*2*^_*p*_ = 0.003], Central [*F*(1, 15) = 0.286, *p* = 0.60, *Ƞ*^*2*^_*p*_ = 0.019] and Right Region [*F*(1, 15) = 0.67, *p* = 0.43, *Ƞ*^*2*^_*p*_ = 0.04]. However, there was a significant effect of Recording Time for the Left [*F*(1, 15) = 17.86, *p* = 0.001, *Ƞ*^*2*^_*p*_ = 0.54], Central [*F*(1, 15) = 17.785, *p* < 0.001, *Ƞ*^*2*^_*p*_ = 0.54] and Right Region [*F*(1, 15) = 25.398, *p* = 0.001, *Ƞ*^*2*^_*p*_ = 0.58], with an increase in amplitude between pre- and post-stimulation EEG in all the three electrode Regions (Fig. 6). Despite the effect of the Recording Time between pre- and post-stimulation EEG, no significant interaction between Stimulation Type and Recording Time was found for Left [*F*(1, 15) = 0.21, *p* = 0.83, *Ƞ*^*2*^_*p*_ = 0.003], Central [*F*(1, 15) = 0.48, *p* = 0.5, *Ƞ*^*2*^_*p*_ < 0.031] and Right Region [*F*(1, 15) = 0.669, *p *= 0.42, *Ƞ*^*2*^_*p*_ = 0.04].

**Fig. 7:**
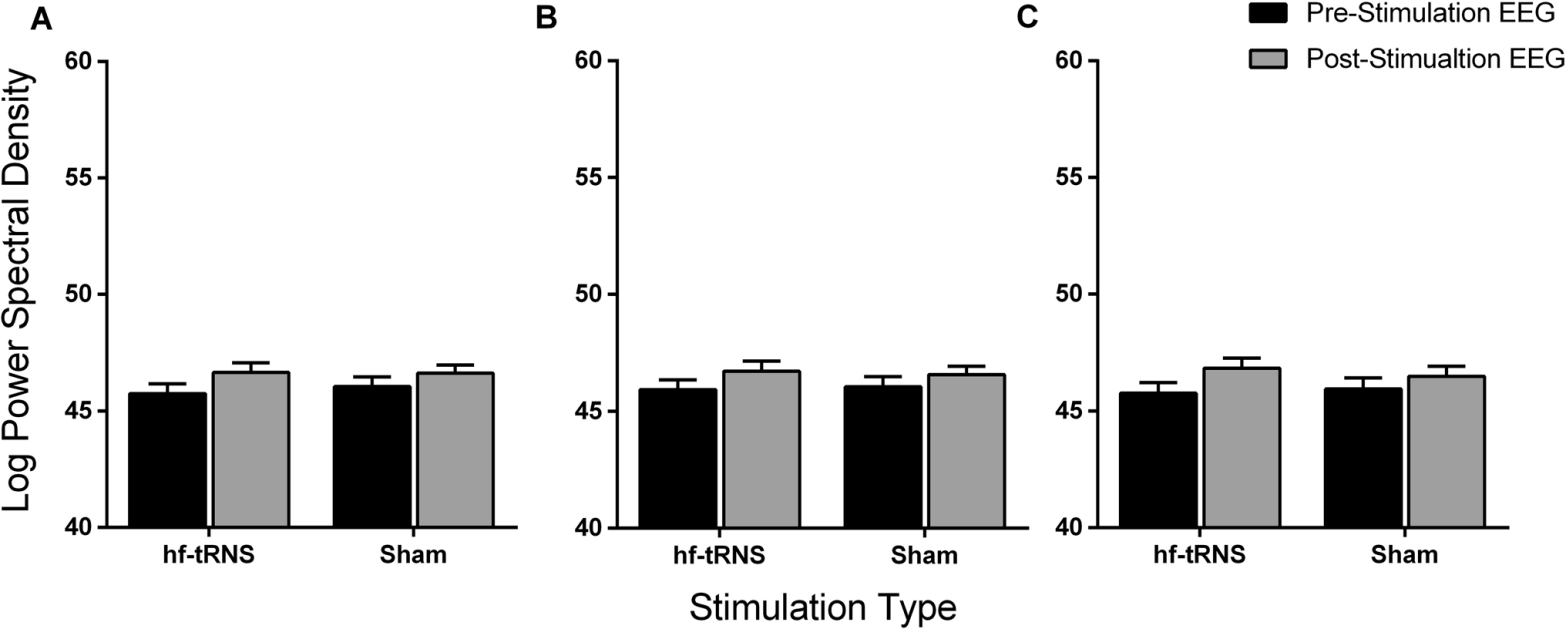
Mean beta power spectral density (PSD) for Left (**A**), Central (**B**), and Right (**C**) Regions. Each Region includes mean PSD values for hf-tRNS and Sham stimulation condition for pre-stimulation EEG (black bars) and post-stimulation EEG (grey bars). Error bars ± SEM

Similarly, for the beta band, the repeated-measures ANOVA did not reveal a significant effect of the Stimulation Type for the Left [*F*(1, 15) = 0.532 , *p* = 0.48, *Ƞ*^*2*^_*p*_ = 0.034], Central [*F*(1, 15) = 0.03, *p* = 0.96, *Ƞ*^*2*^_*p*_ < 0.001], and Right Region [*F*(1, 15) = 0.088, *p* = 0.77, *Ƞ*^*2*^_*p*_ = 0.006]. However, there was a significant effect of Recording Time for the Left [*F*(1, 15) = 19.08, *p* = 0.001, *Ƞ*^*2*^_*p*_ = 0.56], Central [*F*(1, 15) = 25.398, *p* < 0.001, *Ƞ*^*2*^_*p*_ = 0.63] and Right Region [*F*(1, 15) = 19.635, *p* < 0.001, *Ƞ*^*2*^_*p*_ = 0.57)], with an increase in amplitude between pre- and post-stimulation EEG in all the three electrode Regions (Fig. 7). Moreover, also for the beta oscillations, no significant interaction between Stimulation Type and Recording Time was found for Left [*F*(1, 15) = 1.351, *p* = 0.26, *Ƞ*^*2*^_*p*_ = 0.083], Central [*F*(1, 15) = 1.181, *p* = 0.29, *Ƞ*^*2*^_*p*_ = 0.07] and Right Region [*F*(1, 15) = 1.968, *p* = 0.181, *Ƞ*^*2*^_*p*_ = 0.116].

#### Visual evoked potentials (VEPs)

For the VEPs, the mean amplitude over the time period for each component (P1, N2, and P2) for the electrodes of interest and for each observer was extracted. Data from electrodes were pooled into the three Regions (Left, Central and Right), and these were averaged across all participants. Separated repeated-measures ANOVAs for each component (P1, N2 and P2) and for each Region (Left, Central and Right) were performed including as within-subjects factors Stimulation Type (hf-tRNS and Sham) and Recording Time (pre-stimulation EEG and post-stimulation EEG).

**Fig. 8:**
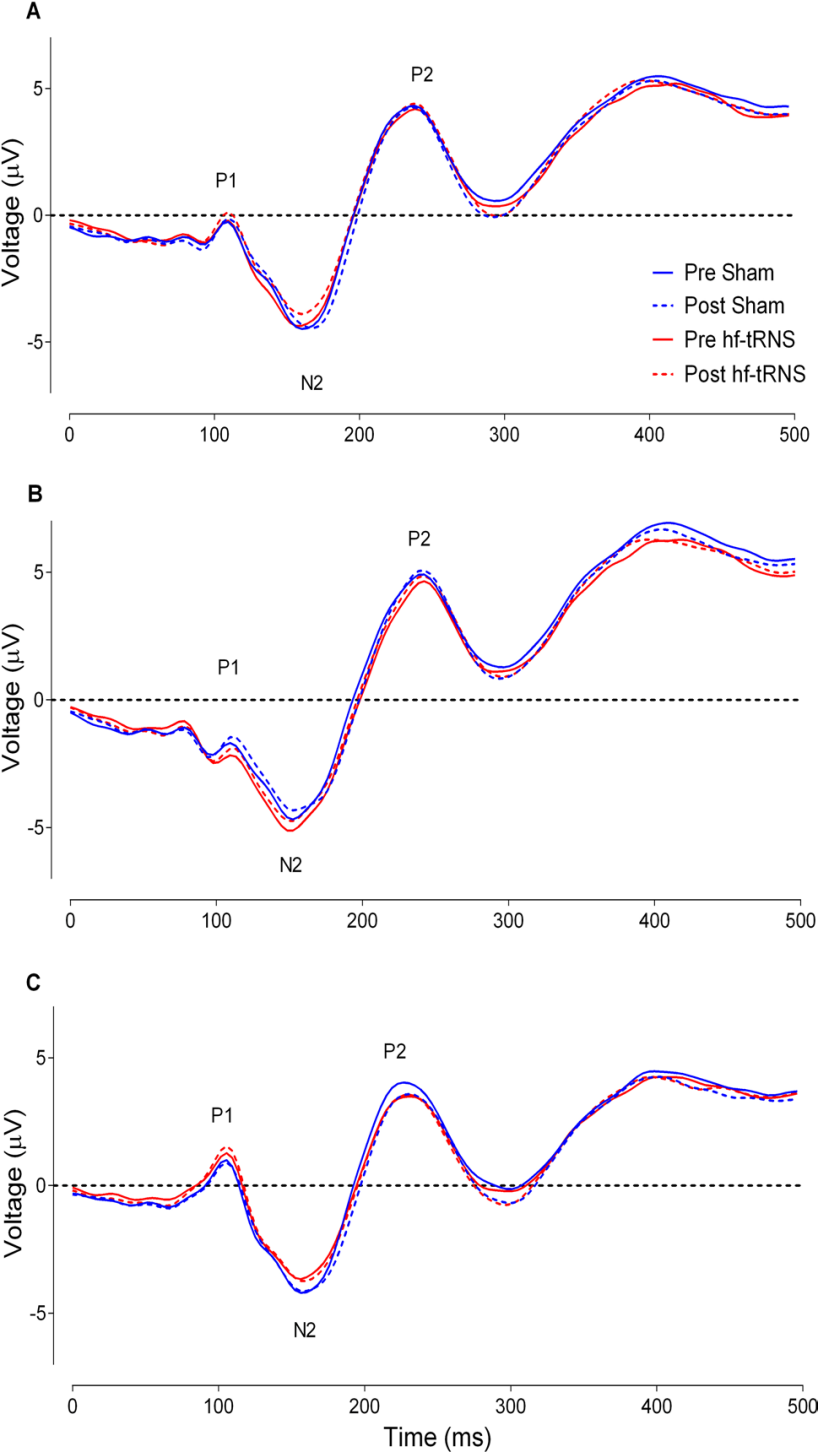
Mean Visual Evoked Potentials (VEP) for the Left (**A**), Central (**B**) and Right (**C**) electrodes. VEPs are illustrated for each stimulation condition (hf-tRNS in red, Sham stimulation in blue) and Recording Time: pre-stimulation EEG (solid lines) and post-stimulation EEG (dashed lines)

Figure 8A shows VEPs for the Left Region. For the Left Region, a Shapiro–Wilk test showed that residuals of mean amplitudes for all the components of interest were normally distributed (*p* > 0.05), with the exception of the P2 component of the post-stimulation EEG in the Sham condition (*p* = 0.027). Figure 8B shows VEPs for the Central electrodes. A Shapiro–Wilk test showed that residuals for mean amplitudes for all the components of interest were normally distributed (*p* > 0.05) except for the P2 component of the post-stimulation EEG in the hf-tRNS condition (*p* = 0.035) and the N2 component of the post-stimulation EEG in the hf-tRNS condition (*p* = 0.044). Figure 8C shows VEPs for the Right electrodes. For the Right electrodes, a Shapiro–Wilk test showed that residuals for mean amplitudes for all the components of interest were normally distributed (*p* > 0.05) apart from the P1 component of the post-stimulation EEG in the Sham condition (*p* = 0.024).

The results of the repeated measure ANOVA for each component (P1, N2 and N2) and for each Region (Left, Central and Right) showed that for all P1, N2 and P2 components in the Left, Central and Right Regions, there was no significant effect of Stimulation Type, Recording Time and interaction between Stimulation Type and Recording Time (all *p* > 0.05, see Table S3 in *Supplementary Material*).

#### Time–frequency analysis

To detect significant changes in event-related spectral perturbation (ERSP) measures, we used the *statcond()* function from EEGLAB (Delorme and Makeig 2004) using paired *t *tests with bootstrapping and correcting the resulting *p* values with the FDR correction (Benjamini and Yekutieli 2001). Bootstrapping is a statistical approach in which a surrogate distribution of data is constructed by selecting spectral estimates from randomly selected samples (with replacement) and then averaging these. We applied this process 150 K times and produced a surrogate baseline data distribution whose specified percentiles are then taken as significance thresholds (*p* < 0.05) (Delorme and Makeig 2004). Therefore, bootstrapping was used to visualise significant deviations from baseline random fluctuations and for testing significant values between conditions (i.e. pre-stimulation and post-stimulation for the Sham and hf-tRNS condition).

The ERSP analysis measures brain oscillation activity changes as a function of time relative to an event. Therefore, ERSP can detect localised time-locked amplitude spectrum increments (i.e. synchronisation) and decrements (i.e. desynchronization) in specific frequency ranges (Makeig 1993; Pfurtscheller 1992). Here, ERSP values in the time–frequency map are plotted in decibels (dB) and normalised and scaled for the rest of the epoch. The comparison between pre- and post-stimulation shows ERSP values in decibels (dB). The levels of dB represent increase or decrease in spectral EEG power. Importantly, a positive difference between pre- and post-stimulation phase represents reduced ERSP power in the post- compared to the pre-stimulation phase. On the other hand, negative values indicate increased ERSP power in the post- compared to the pre-stimulation phase. Similarly, when ERSP difference between post-stimulation Sham and post-stimulation hf-tRNS is positive this represents reduced ERSP in the post-stimulation hf-tRNS compared to the post-stimulation Sham, whereas negative values indicate an increased ERSP in the post-stimulation hf-tRNS compared to the post-stimulation Sham.

**Fig. 9:**
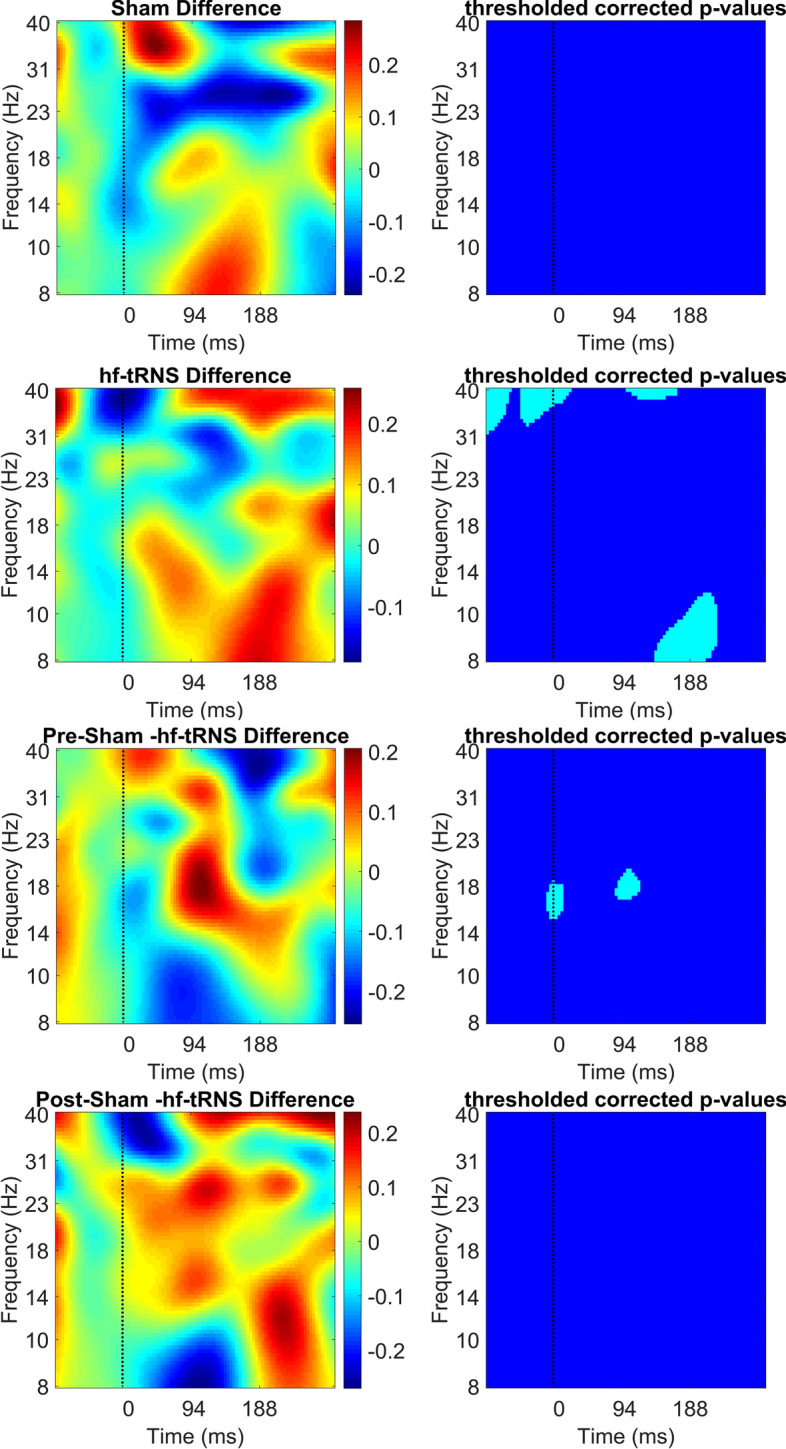
Difference in ERSP (in dB) between pre- and post-stimulation EEG for the Sham condition (first and second panel), for the hf-tRNS condition (third and fourth panel), for the pre-stimulation EEG Sham and hf-tRNS condition (fifth and sixth panel), and for post-stimulation EEG in the Sham and hf-tRNS condition (seventh and eighth panel) for the Left Region (electrodes: O1, PO7, P7, P5, P3, P1). Statistically significant *p *values (after FDR correction) are indicated as cyan, non-statistically significant *p* values are indicated as dark blue. The vertical dashed line at 0 ms represents the stimulus onset. The upper end of the colour bar (red) indicates event-related desynchronization while bottom end of the colour bar (blue) indicates synchronization

**Fig. 10:**
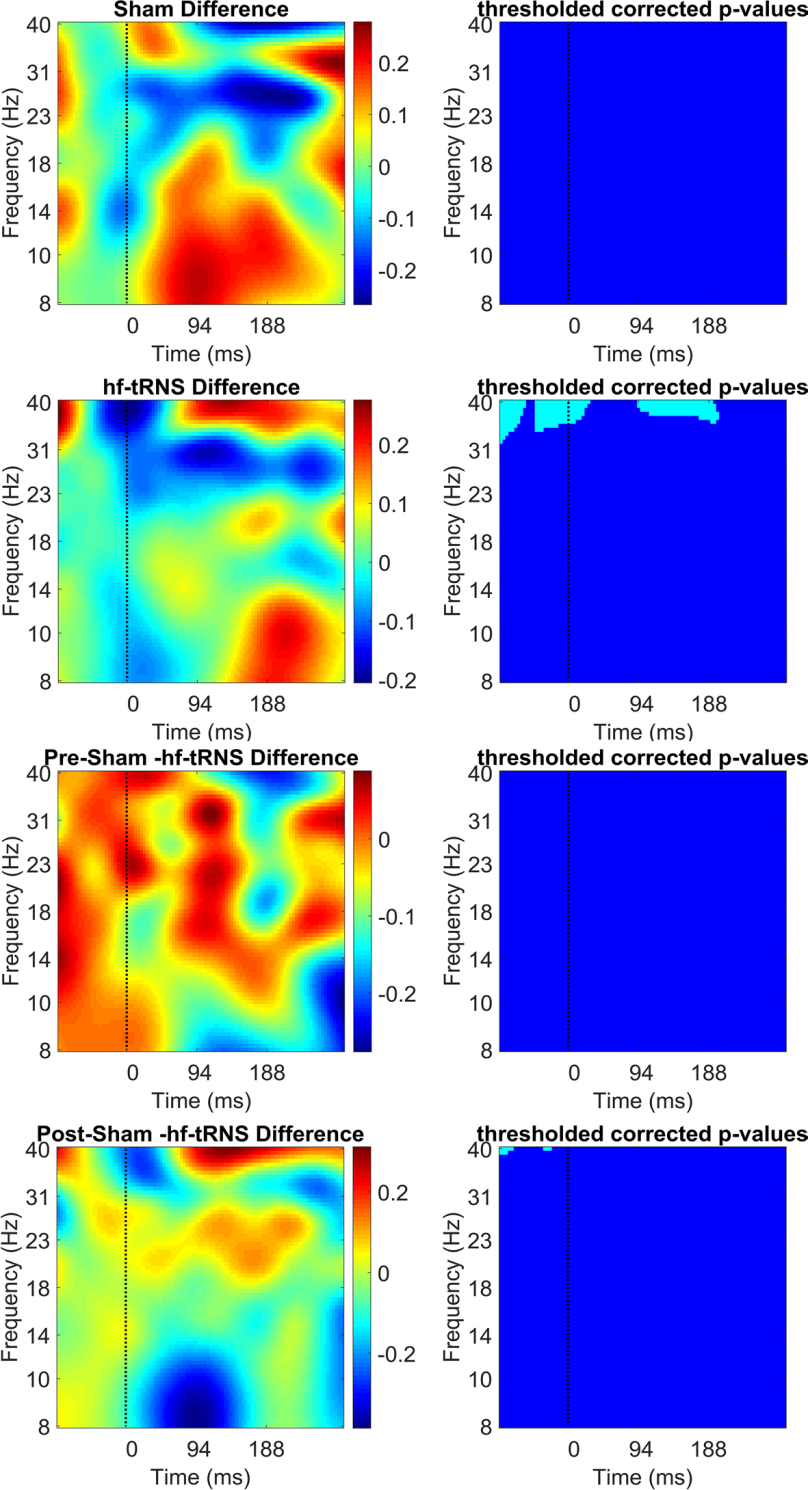
Difference in ERSP (in dB) between pre- and post-stimulation EEG for the Sham condition (first and second panel), for the hf-tRNS condition (third and fourth panel), for the pre-stimulation EEG Sham and hf-tRNS condition (fifth and sixth panel), and for post-stimulation EEG in the Sham and hf-tRNS condition (seventh and eighth panel) for the Central Region (electrodes: Oz, POz, Pz). Statistically significant *p* values (after FDR correction) are indicated as cyan, non-statistically significant *p* values are indicated as dark blue

**Fig. 11:**
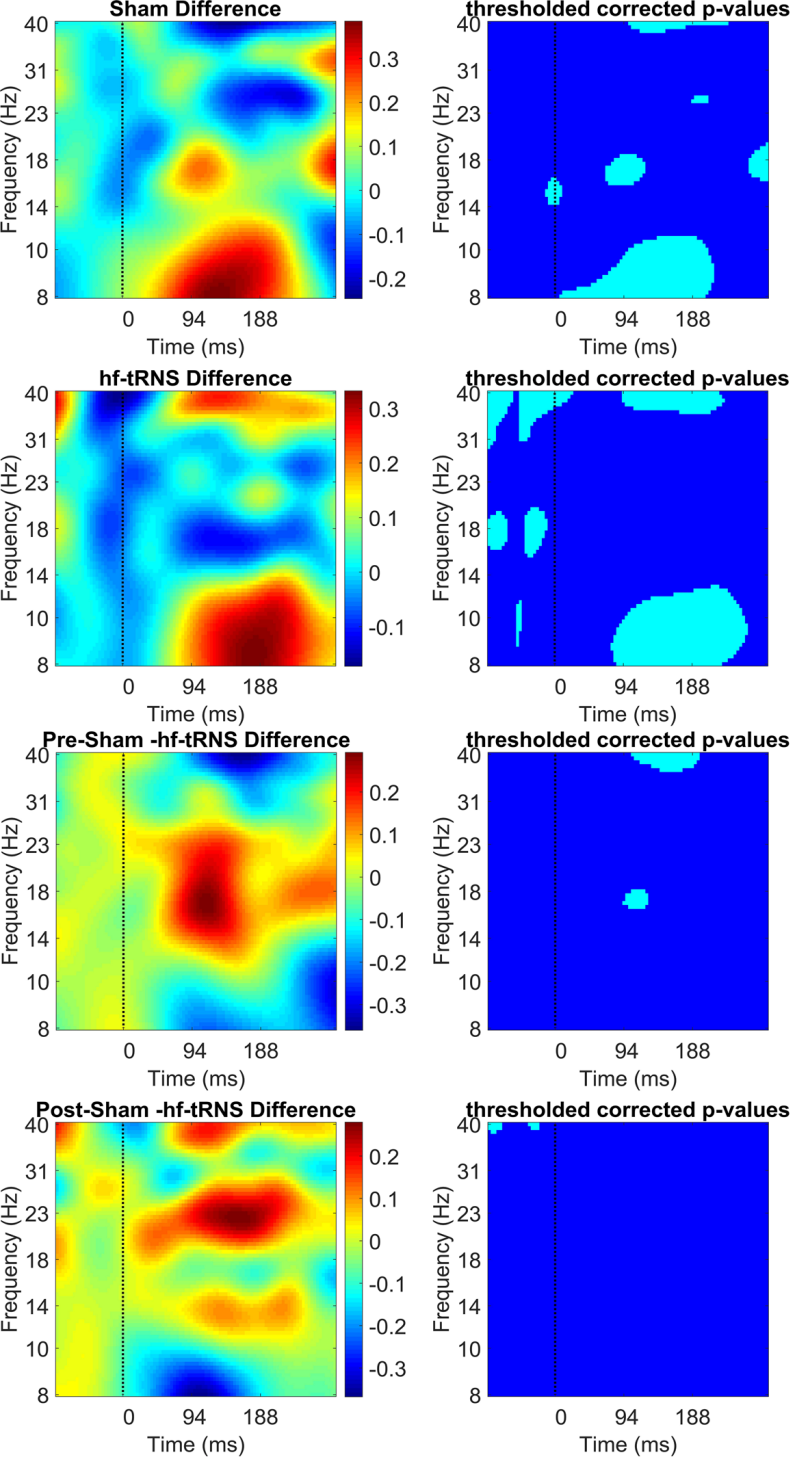
Difference in ERSP (in dB) between pre- and post-stimulation EEG for the Sham condition (first and second panel), for the hf-tRNS condition (third and fourth panel), for the pre-stimulation EEG Sham and hf-tRNS condition (fifth and sixth panel), and for post-stimulation EEG in the Sham and hf-tRNS condition (seventh and eighth panel) for the Right Region (electrodes: O2, PO8, P8, P6, P4, P2). Statistically significant *p* values (after FDR correction) are indicated as cyan, non-statistically significant *p* values are indicated as dark blue

**Fig. 12:**
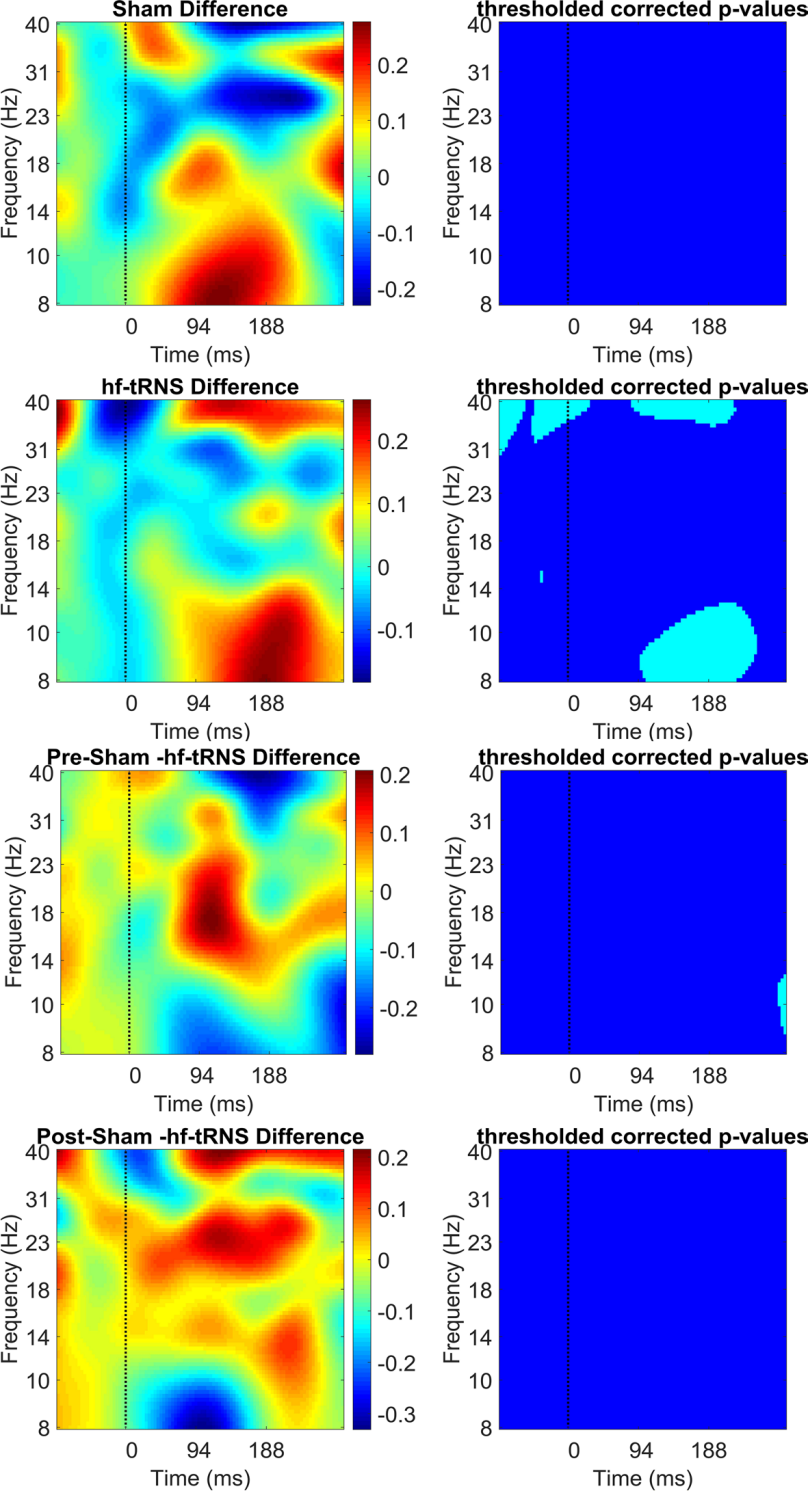
Difference in ERSP (in dB) between pre- and post-stimulation EEG for the Sham condition (first and second panel), for the hf-tRNS condition (third and fourth panel), for the pre-stimulation EEG Sham and hf-tRNS condition (fifth and sixth panel), and for post-stimulation EEG in the Sham and hf-tRNS condition (seventh and eighth panel) averaged for all the electrodes (i.e. O1, PO7, P7, P5, P3, P1, Oz, POz, Pz, O2, PO8, P8, P6, P4, and P2). Statistically significant *p* values (after FDR correction) are indicated as cyan, non-statistically significant *p* values are indicated as dark blue

Figure 9 shows ERSP power results of the time–frequency decomposition averaged over the channels O1, PO7, P7, P5, P3, P1 (i.e. Left Region) as a difference between pre- and post-stimulation EEG separately for Sham and hf-tRNS condition (first to fourth panel), and the difference between pre-stimulation EEG Sham and pre-stimulation EEG hf-tRNS and between post-stimulation EEG Sham and post-stimulation EEG hf-tRNS (fifth to eighth panels). The upper end of the colour bar (red) indicates event-related decrement in spectral power (i.e. desynchronization), while bottom end (blue) of the colour bar indicates event-related increment in spectral power (i.e. synchronisation). The same applies to Figs. 10, 11, 12.

No significant changes in ERSP power between pre- and post-stimulation EEG in the Sham condition was found. The difference between pre- and post-stimulation EEG in the hf-tRNS condition showed reduced ERSP power in the pre-stimulus onset (from − 100 to − 50 ms) and post-stimulus onset (from 100 to 150 ms) in the high beta (20–28 Hz) and low gamma band (30–40 Hz). Moreover, an increase in ERSP power in the difference pre–post-stimulation EEG in the hf-tRNS condition in the low gamma band, was found shortly before and after stimulus onset (from − 50 to 20 ms). Reduced ERSP power around 150–220 ms post-stimulus in the alpha band (8–14 Hz) was also found. The difference between pre-stimulation EEG between Sham and hf-tRNS condition revealed a limited but significant increase of the ERSP power around the onset of the stimulus, followed by a reduced ERSP power around 100 ms after the onset of the stimulus in the beta band (15–30 Hz). A comparison between post-stimulation EEG in Sham and hf-tRNS conditions did not show any significant change in ERSP power.

Figure 10 shows ERSP power results averaged over the channels Oz, POz and Pz (i.e. Central Region). As in Fig. 9, ERSP are plotted as a difference between pre–post-stimulation EEG separately for the Sham and hf-tRNS condition (first to fourth panel), and the difference between post-stimulation EEG Sham and post-stimulation EEG hf-tRNS (fifth and sixth panels). In the Central Region, no significant changes in ERSP power were found between pre- and post-stimulation EEG in the Sham condition. The difference between pre- and post-stimulation EEG in the hf-tRNS condition showed reduced ERSP power in the pre-stimulus onset epoch (from − 100 to − 50 ms) and in the post-stimulus onset epoch (from 90 to 190 ms) around high beta (20–28 Hz) and low gamma band (30–40 Hz). On the other hand, increased ERSP power was found in the same frequency range between pre-stimulus onset epoch and post-stimulus onset epoch (from − 50 to 20 ms). No significant difference was found between pre-stimulation EEG in the Sham and in the hf-tRNS condition. The comparison between post-stimulations EEG for Sham and for hf-tRNS conditions resulted in a significant reduced ERSP power at 40 Hz before the stimulus onset (− 100), followed by increase in ERSP power shortly before the stimulus onset (− 20 ms).

Figure 11 shows ERSP power results of the time–frequency decomposition averaged over the electrodes O2, PO8, P8, P6, P4, P2 (i.e. Right Region). As in Figs. 9 and 10, ERSP are plotted as a difference between pre–post-stimulation EEG separately for the Sham and hf-tRNS condition (first to fourth panel), and the difference between post-stimulation EEG in the Sham and post-stimulation EEG in hf-tRNS conditions (fifth and sixth panels).

The difference between pre- and post-stimulation EEG in the Sham condition showed a significant increase in ERSP power in the low gamma band (40 Hz) in the post-stimulus onset epoch (from 90 to 230 ms). There was also a significant increase in ERSP power in the beta band (14–30 Hz) at stimulus onset, followed by a decrease in ERSP power in the post-stimulus onset epoch (from 90 to 100 ms and from 250 to 300 ms). A significant decrease in ERSP power in the difference between pre–post-stimulation EEG in the Sham condition was also found in the alpha band (8–14 Hz) in the post-stimulus onset epoch (90–200 ms).

The difference between the pre- and post-stimulation EEG in the hf-tRNS condition showed a significant reduction in ERSP power in the pre-stimulus onset epoch (from − 100 to − 50 ms) and post-stimulus onset epoch (from 100 to 150 ms) in the high beta (20–28 Hz) and low gamma band (30–40 Hz). In addition, a significant increase in ERSP power starting in pre-stimulus onset and lasting shortly in the post-stimulus onset epoch (from − 50 to 20 ms) was found. Reduced ERSP power in the early pre-stimulus onset epoch (from − 100 to − 50 ms) followed by increase in ERSP values (from − 50 to − 20 ms) was also found in the beta band (15–30 Hz). Furthermore, we found a significant decrease in ERSP power in the alpha band post-stimulus onset epoch (from 90 to 250 ms). The difference between pre-stimulation EEG between Sham and hf-tRNS condition revealed a significant increase in ERSP power between 100 and 190 ms in the low gamma band (40 Hz). Furthermore, as for the Left region, we found a small significant decrease around 100 ms in the beta band (15–30 Hz). The comparison between post-stimulations EEG for Sham and for hf-tRNS conditions resulted in a significant reduced ERSP power at 40 Hz before the stimulus onset (− 100), followed by increase in ERSP power shortly before the stimulus onset (−20 ms).

To investigate the effects of electrical stimulation on the parieto-occipital cortex as a whole, the ERSP values of the three ROIs where averaged over (i.e. Left, Right and Central regions). Figure 12 shows the results of the time–frequency decomposition averaged over all the electrode of interests (i.e. O1, PO7, P7, P5, P3, P1, Oz, POz, Pz, O2, PO8, P8, P6, P4, and P2). Again, as in Figs. 9, 10, 11. ERSP power results are plotted as a difference between pre–post-stimulation EEG separately for the Sham and hf-tRNS condition (first to fourth panel), and the difference between post-Sham and post-hf-tRNS (fifth and sixth panels). No significant changes in ERSP power were found for the difference between pre- and post-stimulation EEG in the Sham condition. However, in the comparison between pre- and post- stimulation EEG in the hf-tRNS condition, we found a reduction ERSP values in the pre-stimulus (from − 100 to − 50 ms) and post-stimulus epoch (from 100 to 200 ms) and an increase in ERSP power starting before and lasting shortly after the stimulus onset (from − 50 to 20 ms) in low gamma band (30–40 Hz). Furthermore, there was a significant decrease in ERSP power in the alpha band after stimulus onset (from 95 to 210 ms). A small increase in ERSP power in the alpha band (8–14 Hz) was found in the pre-stimulation EEG between Sham and hf-tRNS, around 300 ms from the stimulus onset. Finally, no significant changes in ERSP power were found in the difference between post-stimulation EEG in the Sham and hf-tRNS condition.

## Discussion

We tested whether a single hf-tRNS session delivered *offline* induced physiological modulation of activity in the visual cortex. Participants performed a direction discrimination task between two random dot kinematograms (RDKs) presented in two distinct temporal intervals. hf-tRNS-induced aftereffects were assessed both at the behavioural level, and by measuring brain activity. This activity consisted of oscillations at rest, amplitude of motion-related visual evoked potentials (VEPs), and event-related spectral perturbation (ERSP). Electrophysiological data were averaged across multiple electrodes to determine three ROIs (left: O1, P1, P3, P5, P7; central; Oz, Pz, Cz and right: O2, P2, P4, P6, P8).

### Effects of offline hf-tRNS on behavioural performance

The results showed that *offline* hf-tRNS did not modulate performance at the motion direction discrimination task. This is the case with respect to the pre-stimulation EEG, and with respect to the Sham stimulation. Therefore, hf-tRNS aftereffects on the visual cortex could be insufficiently strong or long-lasting to modulate the mechanism involved in the motion perception task. Previous findings also showed limited effects of offline stimulation compared to *online* hf-tRNS in improving performance in an orientation discrimination task (Pirulli et al. 2013).

Previous study demonstrated that *online* hf-tRNS was able to improve visual motion perception with near threshold stimuli (~ 60% correct performance) boosting the activity of neurons near the firing threshold possibly synchronising their activity (Pavan et al. 2019). This modulation has been explained within the stochastic resonance framework. Stochastic resonance is a phenomenon resulting from the combination of a threshold, a subthreshold stimulus and noise. Normally, a stimulus can be encoded and perceived when it crosses a given threshold. However, if the stimulus is subthreshold, its probability of crossing the threshold is low. Provided a non-linear system like the brain, if an “optimal” amount of noise is applied to the system (e.g. using hf-tRNS) this might lead to an enhanced signal (McDonnell and Abbott 2009; Ward 2009). In contrast to Pavan et al. (2019), the current study employed an *offline* hf-tRNS protocol, whose mechanisms of action are still debated. In addition, the current study employed a higher coherence threshold (~ 80%) with respect to the study of Pavan et al. (2019). The higher coherence threshold used might have hindered the possibility to induce robust modulation of the behavioural performance. On the other hand, high coherence threshold (~ 80%) was used to induce robust stimulus-locked electrophysiological responses (Niedeggen and Wist 1998; Patzwahl and Zanker 2000). In general, it should be noted that while *online* hf-tRNS increases performance when stimuli are presented at near threshold (~ 60%), more experiments are necessary to assess whether the same modulation would occur with the same coherence level and with an *offline* stimulation protocol.

### Effects of offline hf-tRNS on PSD at rest

The electrophysiological results showed that alpha (8–14 Hz) and beta (15–30 Hz) band oscillations at rest significantly increased power spectral density (PSD) on the post-stimulation EEG with respect to the pre-stimulation EEG. Furthermore, this increase was not restricted to a specific location, but was present for all the electrode Regions considered. In addition, no significant difference was found for delta and theta bands regardless of the stimulation condition.

The level of alpha power at rest is linked to cortical excitation and metabolic activity; larger alpha power is thought to be indicative of synchronised activity and reduced metabolic activation (Nunez and Silberstein, 2000). The increased synchronisation in the alpha band post-stimulation EEG with respect to the pre-stimulation EEG might be due to an enhanced relaxation state or increased fatigue of the observers during the testing session. The results for the beta oscillations also showed a similar trend. In fact, the PSD was significantly higher in the post-stimulation EEG with respect to the pre-stimulation EEG in both Sham and hf-tRNS conditions. Few studies showed positive correlations for alpha and beta bands at rest with metabolic activity in areas associated to the default model network (DMN) such as temporal-parietal junction, inferior parietal junction, and frontal gyrus (Laufs et al. 2003; Mantini et al. 2007). Overall, the results showed that 20 min of *offline* hf-tRNS delivered over the parieto-occipital cortex are not able to modulate brain activity at rest.

### Effects of offline hf-tRNS on VEPs

The motion direction discrimination task employed in this study successfully elicited several VEP components related to visual motion processing, such as P1, N2 and P2. However, statistical analysis showed no significant aftereffects induced by *offline* hf-tRNS on amplitude for any of the VEP components when compared to the pre- and post-stimulation EEG phases or between Sham and hf-tRNS conditions. Although inclusive, to our knowledge this was the first study investigating the effects of *offline* hf-tRNS on VEPs and these results might pose some limitation of the strength of the aftereffects of this stimulation on VEP modulation, when using supra-threshold coherence stimuli. Future studies should, therefore, investigate the possibility to induce VEP changes in combination with different stimulation protocols (i.e. *online* hf-tRNS) and different motion coherence thresholds for the stimuli used.

### Effects of offline hf-tRNS on evoked response spectral perturbation (ERSP)

Time–frequency decomposition showed significant differences in evoked response spectral perturbation (ERSP) for post-stimulation EEG with respect to pre-stimulation EEG. Significant differences were found between pre- and post-stimulation EEG in hf-tRNS condition in all three regions, but also for the Sham condition in the right region. Furthermore, significant differences in ERSP power were also found when considering the data of all the electrodes of interest, but only for the hf-tRNS condition. Finally, a small but still significant difference in ERSP power was also found between post-stimulation Sham and post-stimulation hf-tRNS for the central and right regions. Specifically, the difference between pre- and post-stimulation ERSP power in the gamma band was characterised by the following pattern: a lower post-stimulation ERSP around 100 and 80 ms before the stimulus onset, followed by an increased ERSP 50 ms before the stimulus onset and lasting until 20–30 ms after the stimulus onset, then another decrease in ERSP around 100–200 ms after the stimulus onset. Importantly, these changes in ERSP in the low gamma band were evident only in the hf-tRNS condition in all the three regions (Figs. 9, 10, 11) and when considering all the electrodes of interest (Fig. 12). However, in the Sham condition only a decrease in ERSP after the stimulus onset was found in the right region and in a narrow band of the spectrum (i.e. around 40 Hz). In addition, the comparison between post-stimulation Sham and hf-tRNS conditions showed a significant difference in ERSP only for the central and right regions. It is worth noting that the difference between Sham and hf-tRNS in the post-stimulation condition showed a decrease-increase pattern of ERSP in the pre-stimulus onset epoch in the gamma band (see, seventh panel in Figs. 10 and 11, respectively), that was also present in the pre- and post-stimulation ERSP difference in the hf-tRNS condition (third panel in Figs. 10 and 11, respectively). Gamma band neural oscillations have been associated with cognitive processes such has perception, attention and memory and it has been suggested to increase visual perception performance (Herrmann et al. 2010). For instance, it is well established that an increase in gamma synchronisation occurs when a sensory stimulus is presented or in correspondence of the cognitive process under investigation (Sedley and Cunningham 2013). Furthermore, increases in gamma oscillatory activities have been associated with attention and stimulus expectancy until its appearance (Engel et al. 2001; Hanslmayr et al. 2007; Von Stein et al. 2000). While changes in the gamma band may indicate differences between the pre- and post-stimulation in the hf-tRNS, the small area of statistically significant differences between post-stimulation Sham and hf-tRNS conditions limit these findings. Therefore, more evidence is needed to understand the possible mechanisms underlying the aftereffect of hf-tRNS on ERSP power. The comparison between pre- and post-stimulation for the right region showed also significant changes in ERSP in the middle range of the beta band (i.e. 16–20 Hz). Similarly, to the gamma band, a transition from decrease to increase ERSP was evident before the stimulus onset and around the stimulus onset. However, both these changes seemed not be affected by the stimulation condition demonstrating that offline hf-tRNS did not affect ERSP in the beta bands. Finally, we also found a significant decrease in ERSP in the post-stimulus onset for the alpha frequency range between the pre- and post-stimulation conditions. Specifically, this was found for the left and right regions and for the parieto-occipital cortex (i.e. averaging over the left, right and central ROIs). While this decrease was present for both stimulation conditions (i.e. Sham and hf-tRNS) in the right region, it was only present for the hf-tRNS condition in the left region and for the posterior cortex. Finally, the comparison between Sham and hf-tRNS at baseline showed significant differences in ERSP power in delimited time–frequency windows. The differences were localised in both left and right regions around the middle range of the beta band (i.e. 16–20 Hz), for the right region in the gamma band (40 Hz), and in the alpha band (8–14 Hz) when considering all the electrodes averaged. These differences were unexpected since we used a within-subject design, an offline stimulation protocol, and a randomised order of the stimulation conditions to avoid any carry over effect of the stimulation (e.g. when hf-tRNS was delivered in the first session). We can exclude the possibility that baseline differences may depend on task-related learning effects. In fact, participants were trained to perform the task before the EEG recording and at the beginning of each experimental session motion coherence threshold were estimated individually for each participant (phase 1 and 2). In addition, it should be noted that increase and decrease patterns of ERSP power found in the baseline (i.e. pre-stimulation EEG Sham and hf-tRNS condition) are not present in the other comparisons (i.e. pre- and post-stimulation EEG for the Sham and hf-tRNS conditions, and for post-stimulation EEG in the Sham and hf-tRNS conditions). This holds for all the regions but the right region (see Fig. 11), in which the difference between pre-stimulation EEG between Sham and hf-tRNS condition revealed a significant increase in ERSP power between 100 and 190 ms in the low gamma band (40 Hz). However, for the same region, there was a decrease in ERSP power in the low gamma band (40 Hz) around 90–200 ms when considering the pre- and post-stimulation EEG for the Sham and hf-tRNS conditions. Given that there was not a clear pattern of increase/decrease of ERSP power between baselines (i.e. pre-stimulation EEG Sham and hf-tRNS condition), we ascribed the reported ERSP baseline differences to a stochastic emergence of unspecified noise possibly introduced by physiological (i.e. metabolic) and/or psychological (e.g. attention and alertness) factors between the experimental sessions that could have influenced the EEG activity (Cacot et al. 1995; Cummings et al. 2000; Ly et al. 2016).

Overall, our results show that *offline* hf-tRNS might induce some modulation of the gamma oscillatory activity at different time points before and after the stimulus onset. This pattern was measured in the pre- and post-stimulation hf-tRNS comparisons but was only found for a marginal time–frequency area in the comparison between post-stimulation EEG in the Sham and hf-tRNS condition. On the other hand, *offline* hf-tRNS does not seem to affect neither alpha nor beta bands.

### Limitations and conclusion

Limitations of this study should also be considered. For example, the number of missing electrodes for the left region was higher with respect to the right region, for which there were no missing electrodes. This was certainly due to technical issues with the recording equipment. Therefore, we cannot exclude the possibility that the activity of these missing electrodes could have affected the results for the left region. In conclusion, the results of the present study showed that one session of *offline* hf-tRNS delivered bilaterally over the parieto-occipital cortex did not produce any effect on VEP amplitude and power of band oscillations at rest. Interestingly, we found significant modulations of ERSP between pre- and post-stimulation EEG hf-tRNS gamma bands and limited, but significant, modulation between pre- and post-stimulation EEG in the Sham and hf-tRNS conditions. Effects within the gamma band appear to be consistent across all the electrodes of interest, therefore, comprising the whole parieto-occipital cortex. On the other hand, the small differences found when comparing post-stimulation Sham and hf-tRNS poses some limit to the efficacy of *offline* stimulation protocols in modulating cortical activity. These results suggest that hf-tRNS aftereffects are highly dependent on the type of stimulation paradigm employed and on the complexity of the task used. We acknowledge that more studies are necessary to better understand the underlying physiological effects of improved behavioural performance following a single session of hf-tRNS.

## Supplementary Information

Below is the link to the electronic supplementary material.

Supplementary file1 (DOCX 30 kb)

## Data Availability

The approved Ethics for this study does not consider and allow data sharing on publicly available research data repositories. Therefore, data are available upon request to the corresponding author.
