# Supplementary material for: Electrophysiological aftereffects of high-frequency transcranial random noise stimulation (hf-tRNS): an EEG investigation
Source: Exp Brain Res. 2021 Jun 8;239(8):2399–418. doi: 10.1007/s00221-021-06142-4 (PMC8354881; doi:10.1007/s00221-021-06142-4)
Supplement: Supplementary file 1 — Supplementary file1 (DOCX 30 kb) [file 221_2021_6142_MOESM1_ESM.docx]

|  |  | | | |
| --- | --- | --- | --- | --- |
| **Frequency band (Hz)** | **Statistics** | | | |
|  |  | ***F_(1,16)_*** | ***p*** | ***Ƞ^2^ _p_*** |
| **Delta (2-4 Hz)** | Stimulation Type | 0.720 | 0.41 | 0.046 |
|  | Recording Time | 0.403 | 0.535 | 0.026 |
|  | Region | **8.508** | **0.001*** | **0.362** |
|  | Stimulation Type x Recording Time | 0.838 | 0.374 | 0.053 |
|  | Stimulation Type x Region | 0.622 | 0.544 | 0.040 |
|  | Stimulation Time x Region | 0.469 | 0.630 | 0.30 |
|  | Stimulation Type x Recording Time x Region | 1.109 | 0.343 | 0.069 |
| **Theta (4-8 Hz)** | Stimulation Type | 0.03 | 0.865 | 0.002 |
|  | Recording Time | 2.596 | 0.128 | 0.148 |
|  | Region | **17.738** | **<0.001*** | **0.542** |
|  | Stimulation Type x Recording Time | 0.793 | 0.387 | 0.50 |
|  | Stimulation Type x Region | 0.731 | 0.490 | 0.46 |
|  | Stimulation Time x Region | 0.231 | 0.795 | 0.015 |
|  | Stimulation Type x Recording Time x Region | 0.399 | 0.674 | 0.026 |
| **Alpha (8-14Hz)** | Stimulation Type | 0.351 | 0.562 | 0.023 |
|  | Recording Time | **19.722** | **<0.001*** | **0.568** |
|  | Region | **8.921** | **0.001*** | **0.373** |
|  | Stimulation Type x Recording Time | 0.457 | 0.509 | 0.030 |
|  | Stimulation Type x Region | 0.601 | 0.555 | 0.039 |
|  | Stimulation Time x Region | 2.219 | 0.126 | 0.129 |
|  | Stimulation Type x Recording Time x Region | 0.857 | 0.434 | .054 |
| **Beta (15-30 Hz)** | Stimulation Type | 0.005 | 0.942 | <0.001 |
|  | Recording Time | **22.856** | **<0.001*** | **0.604** |
|  | Region | 0.041 | 0.960 | 0.003 |
|  | Stimulation Type x Recording Time | 1.621 | 0.222 | 0.098 |
|  | Stimulation Type x Region | 0.700 | 0.505 | 0.045 |
|  | Stimulation Time x Region | 1.336 | 0.278 | 0.082 |
|  | Stimulation Type x Recording Time x Region | 1.727 | 0.209 | 0.103 |
| **FDR corrected *paired sampled T-Test* for Recording Time** | | | | |
|  | **pre-stimulation EEG** | **Post-stimulation EEG** | ***t-test*** | ***Corrected-p*** |
| **Alpha (8-14Hz)** | *M*=54.26, *SD=*2.17, | *M*=55.16, *SD*=2.22 | -4.44 | **<0.001*** |
| **Beta( 15-30 Hz)** | *M*=45.92, *SD*=1.59, | *M*=46.65, *SD*=1.43 | -4.78 | **<0.001*** |
| **FDR corrected *paired sampled T-Test* for Region** | | | | |
|  | ***Region*** | ***t-test*** | | ***Corrected-p*** |
| **Delta** | *Centre: M*=57.94, *SD=*1.39  *Left: M*=57.41, *SD=*1.35  *Right: M*=57.15, *SD=*1.20 | *Centre-Left:* 4.32  *Centre-Right:* 3.38  *Left-Right:*1.22 | | **0.003***  **0.006***  0.240 |
| **Theta** | *Centre: M*=55.19, *SD=*2.11  *Left: M*=54.58, *SD=*2.10  *Right: M*=53.92, *SD=*1.71 | *Centre-Left:* 3.60  *Centre-Right:* 4.73  *Left-Right:*3.49 | | **0.003***  **<0.001***  **0.003*** |
| **Alpha** | *Centre: M*=55.18, *SD=*2.33  *Left: M*=57.89, *SD=*2.55  *Right: M*=54.05, *SD=*1.80 | *Centre-Left:* 1.09  *Centre-Right:* 4.17  *Left-Right:*2.84 | | 0.294  **0.003***  **0.018*** |

**Table S1.** Summary of statistics for PSD in the delta, theta, alpha and beta bands.

| ***Left Region*** |  | | | | | | |
| --- | --- | --- | --- | --- | --- | --- | --- |
| **Frequency band (Hz)** | **Statistics** | | | | | | |
|  |  | ***F_(1,15)_*** | | ***p*** | | ***Ƞ^2^ _p_*** | |
| **Delta**  **(2-4 Hz)** | Stimulation Type | 0.302 | | 0.591 | | 0.02 | |
|  | Recording Time | 1.052 | | 0.321 | | 0.07 | |
|  | Stimulation Type x Recording Time | 0.179 | | 0.679 | | 0.012 | |
| **Theta**  **(4-8 Hz)** | Stimulation Type | 0.034 | | 0.855 | | 0.002 | |
|  | Recording Time | 3.316 | | 0.089 | | 0.181 | |
|  | Stimulation Type x Recording Time | 0.162 | | 0.693 | | 0.011 | |
| **Alpha**  **(8-14Hz)** | Stimulation Type | 0.046 | | 0.833 | | 0.003 | |
|  | Recording Time | **17.86** | | **0.001*** | | **0.544** | |
|  | Stimulation Type x Recording Time | 0.21 | | 0.833 | | 0.003 | |
| **Beta**  **(15-30 Hz)** | Stimulation Type | 0.532 | | 0.477 | | 0.034 | |
|  | Recording Time | **19.080** | | **0.001*** | | **0.560** | |
|  | Stimulation Type x Recording Time | 1.351 | | 0.263 | | 0.083 | |
| ***Central Region*** | | | | | | | |
| **Frequency band (Hz)** | **Statistics** | | | | | | |
|  |  | | ***F_(1,15)_*** | | ***P*** | | ***Ƞ^2^ _p_*** |
| **Delta**  **(2-4 Hz)** | Stimulation Type | | 0.085 | | 0.775 | | 0.006 |
|  | Recording Time | | 0.031 | | 0.863 | | 0.002 |
|  | Stimulation Type x Recording Time | | 0.196 | | 0.665 | | 0.013 |
| **Theta**  **(4-8 Hz)** | Stimulation Type | | 0.042 | | 0.840 | | 0.003 |
|  | Recording Time | | 1.193 | | 0.292 | | 0.074 |
|  | Stimulation Type x Recording Time | | 0.399 | | 0.537 | | 0.026 |
| **Alpha**  **(8-14Hz)** | Stimulation Type | | 0.286 | | 0.60 | | 0.019 |
|  | Recording Time | | **17.785** | | **<0.001*** | | **0.542** |
|  | Stimulation Type x Recording Time | | 0.483 | | 0.498 | | 0.031 |
| **Beta**  **(15-30 Hz)** | Stimulation Type | | 0.03 | | 0.954 | | <0.001 |
|  | Recording Time | | **25.398** | | **<0.001*** | | **0.629** |
|  | Stimulation Type x Recording Time | | 1.181 | | 0.294 | | 0.073 |
| ***Right Region*** | | | | | | | |
| **Frequency band (Hz)** | **Statistics** | | | | | | |
|  |  | | ***F_(1,15)_*** | | ***P*** | | ***Ƞ^2^ _p_*** |
| **Delta**  **(2-4 Hz)** | Stimulation Type | | 1.208 | | 0.289 | | 0.75 |
|  | Recording Time | | 0.223 | | 0.644 | | 0.015 |
|  | Stimulation Type x Recording Time | | 1.484 | | 0.242 | | 0.090 |
| **Theta**  **(4-8 Hz)** | Stimulation Type | | 0.40 | | 0.537 | | 0.026 |
|  | Recording Time | | 1.716 | | 0.210 | | 0.103 |
|  | Stimulation Type x Recording Time | | 1.109 | | 0.309 | | 0.069 |
| **Alpha**  **(8-14Hz)** | Stimulation Type | | 0.669 | | 0.426 | | 0.43 |
|  | Recording Time | | **20.824** | | **0.001*** | | **0.581** |
|  | Stimulation Type x Recording Time | | 0.669 | | 0.420 | | 0.044 |
| **Beta**  **(15-30 Hz)** | Stimulation Type | | 0.088 | | 0.771 | | 0.006 |
|  | Recording Time | | **19.635** | | **<0.001*** | | **0.567** |
|  | Stimulation Type x Recording Time | | 1.968 | | 0.181 | | 0.116 |

**Table S2.** Summary of statistics for PSD in the delta, theta, alpha and beta bands separately for Left, Central and Right regions.

| ***Left Region*** |  | | | | | |
| --- | --- | --- | --- | --- | --- | --- |
|  | **Statistics** | | | | | |
| **VEPs** |  | ***F_(1,15)_*** | ***p*** | | ***Ƞ^2^ _p_*** | |
| **P1** | Stimulation Type | 0.790 | 0.39 | | 0.05 | |
|  | Recording Time | 0.120 | 0.73 | | 0.008 | |
|  | Stimulation Type x Recording Time | 0.001 | 0.99 | | 0.001 | |
| **N2** | Stimulation Type | 1.181 | 0.30 | | 0.073 | |
|  | Recording Time | 1.401 | 0.26 | | 0.09 | |
|  | Stimulation Type x Recording Time | 1.404 | 0.25 | | 0.09 | |
| **P2** | Stimulation Type | 0.316 | 0.60 | | 0.021 | |
|  | Recording Time | 3.206 | 0.09 | | 0.176 | |
|  | Stimulation Type x Recording Time | 0.737 | 0.40 | | 0.047 | |
| ***Central Region*** | | | | | | |
|  | **Statistics** | | | | | |
| **VEPs** |  | ***F_(1,15)_*** | | ***p*** | | ***Ƞ^2^ _p_*** |
| **P1** | Stimulation Type | 0.217 | | 0.65 | | 0.014 |
|  | Recording Time | 0.008 | | 0.93 | | 0.001 |
|  | Stimulation Type x Recording Time | 0.032 | | 0.86 | | 0.002 |
| **N2** | Stimulation Type | 0.003 | | 0.95 | | <0.001 |
|  | Recording Time | 0.976 | | 0.34 | | 0.06 |
|  | Stimulation Type x Recording Time | 0.108 | | 0.75 | | 0.007 |
| **P2** | Stimulation Type | 0.021 | | 0.89 | | 0.001 |
|  | Recording Time | 0.029 | | 0.87 | | 0.002 |
|  | Stimulation Type x Recording Time | 0.446 | | 0.51 | | 0.029 |
| ***Right Region*** | | | | | | |
|  | **Statistics** | | | | | |
| **VEPs** |  | ***F_(1,15)_*** | | ***p*** | | ***Ƞ^2^ _p_*** |
| **P1** | Stimulation Type | 2.282 | | 0.15 | | 0.132 |
|  | Recording Time | 0.165 | | 0.69 | | 0.011 |
|  | Stimulation Type x Recording Time | 0.017 | | 0.90 | | 0.001 |
| **N2** | Stimulation Type | 3.928 | | 0.07 | | 0.208 |
|  | Recording Time | 0.018 | | 0.89 | | 0.001 |
|  | Stimulation Type x Recording Time | 0.123 | | 0.731 | | 0.008 |
| **P2** | Stimulation Type | 0.520 | | 0.82 | | 0.003 |
|  | Recording Time | 2.089 | | 0.17 | | 0.122 |
|  | Stimulation Type x Recording Time | 0.304 | | 0.59 | | 0.02 |

**Table S3.** Summary of statistics for amplitude values for P1, N2 and P2 components separately for Left, Central and Right regions.
